# Beyond the horizon: Neutrophils leading the way in the evolution of immunotherapy

**DOI:** 10.1002/cam4.6761

**Published:** 2023-12-07

**Authors:** Sanjana Rajgopal, Kosuke Nakano, Leah M. Cook

**Affiliations:** ^1^ Department of Pathology and Microbiology University of Nebraska Medical Center Omaha Nebraska USA; ^2^ Department of Genetics, Cell Biology, and Anatomy University of Nebraska Medical Center Omaha Nebraska USA; ^3^ Fred & Pamela Buffett Cancer Center Omaha Nebraska USA

**Keywords:** cancer, immunotherapy, neutrophils, tumor microenvironment

## Abstract

Cancer is a complex and dynamic disease, initiated by a multitude of intrinsic mutations and progressed with the assistance of the tissue microenvironment, encompassed by stromal cells including immune cell infiltration. The novel finding that tumors can evade anti‐cancer immune functions shaped the field of immunotherapy, which has been a revolutionary approach for the treatment of cancers. However, the development of predominantly T cell‐targeted immunotherapy approaches, such as immune checkpoint inhibition, also brought about an accumulation of evidence demonstrating other immune cell drivers of tumor progression, such as innate immune cells and notably, neutrophils. In the past decade, neutrophils have emerged to be primary mediators of multiple cancer types and even in recent years, are gaining attention for their potential use in the next generation of immunotherapies. Here, we review current immunotherapy strategies and thoroughly discuss the roles of neutrophils in cancer and novel neutrophil‐targeted methods for treating cancer.

## Graphical abstract

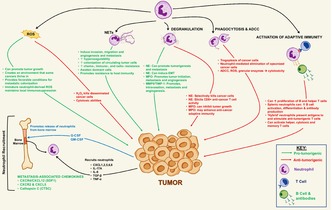

## 1 INTRODUCTION TO CURRENT IMMUNOTHERAPY

One of the hallmarks of cancer originally described by Robert Weinberg and Douglas Hanahan, is the ability of cancer cells to evade an active immune response. Initial carcinogenesis is often driven by an accumulation of genetic mutations that can result in the shedding and release of immune‐activating antigens. However, with cancer progression, tumors promote infiltration of a diverse population of immune cells that can include suppressive innate immune cells and regulatory T cells (promoting “self” antigen recognition) which subsequently block the recruitment and activation of cytotoxic T cells, respectively. Further, the growing tumor releases a collection of immunosuppressive cytokines, further promoting the recruitment and expansion of pro‐tumor and immunosuppressive innate immune cells and/or myeloid‐derived suppressor cells.

Despite the complexity of the tumor microenvironment (TME), immune‐focused therapies have been revolutionary in the fight to improve cancer patient survival. Nearly four decades ago, the field of cancer therapeutics began to shift from cancer‐intrinsic therapies, such as kinase inhibitors, to systemic approaches to target the tumor environment and initiate a systemic anti‐tumor immune response. Although it has long been utilized for therapeutic interventions, immunotherapy quickly developed into feasible cancer‐targeting strategies after the discovery and characterization of T‐cell checkpoint receptors. Immunotherapy has been hugely successful and opens the avenue for more tailored and personalized approaches to expand upon current therapies. However, poor patient response for some cancers along with emerging evidence demonstrating cancer types heavily infiltrated and regulated by myeloid cells, the immunotherapy strategy can no longer be applied as a “one‐size‐fits‐all” approach and requires new considerations about the immune cell microenvironment important for therapeutic efficacy. In this review, we briefly discuss current immunotherapeutic approaches and, for the sake of brevity, neutrophils in cancer, which have been gaining much attention as major drivers of various cancer types as well as promising tools for targeting cancer progression.

## 2 CURRENT IMMUNOTHERAPY APPROACHES

Cancer immunotherapy uses the body's immune system to fight cancer cells rather than relying solely on traditional methods such as chemotherapy and radiation therapy. There are several types of cancer immunotherapy, each targeting and destroying cancer cells in different ways.^1^ The immune system is responsible for identifying and eliminating abnormal cells, including cancer cells. T cells, a type of white blood cell, play a central role in the immune response to cancer. when T cells recognize specific proteins (antigens) on the surface of cancer cells, they initiate an immune response to destroy these malignant cells. However, tumor adaptations that occur with progression including antigen shedding, rapid production of neoantigens, and immune inhibitory cytokine production in the TME can suppress T‐cell recruitment and activation.^2^ A common theme of current immunotherapies is focused on circumventing known inhibitors of T‐cell activation which we briefly discuss.

One type of immunotherapy is adoptive cell transfer (ACT) therapy, which involves removing immune cells from a patient's body, genetically modifying them to recognize and attack cancer cells using cancer‐specific antigens and then reintroducing them into the patient's body. ACT therapy has its roots in early attempts to harness the immune system to fight cancer. In the late 1980s, Steven Rosenberg and colleagues at the National Cancer Institute (NCI) pioneered the isolation of tumor‐infiltrating lymphocytes (TILs) from melanoma patients. They observed that when these TILs were grown and reinjected into patients, the tumors regressed. At the same time, research was also being conducted on lymphocyte‐activated killer (LAK) therapy, in which T cells extracted from the patient's blood were activated with interleukin 2 (IL‐2) and reintroduced into the patient. LAK therapy showed some efficacy, but also had significant side effects.^1^, ^3^

In the 1990s, the concept of gene transfer for immunotherapy gained support. Researchers developed a method to efficiently target cancer cells by genetically engineering T cells to express a specific receptor known as chimeric antigen receptor (CAR). This CAR allows T cells to recognize and bind to specific proteins and antigens on the surface of cancer cells. As a result, CAR T‐cell therapies have been developed and have had remarkable success in certain blood cancers.^4^ Since the 2000s, ACT therapy has continued to evolve with advances in gene editing technologies, such as CRISPR‐Cas9 opening up new possibilities for enhancing T‐cell function and expanding its application to various types of cancers and infectious diseases as well as other immune cells, such as CAR‐M (CAR‐expressing monocytes/macrophages),^5^ CAR‐NK (CAR‐expressing natural killer cells)^6^ and even CAR‐Neu (CAR‐expressing neutrophils).^7^

Another cancer immunotherapy is cancer vaccine therapy. Unlike conventional vaccines used to prevent infectious diseases, cancer vaccines are designed to stimulate the immune system to recognize and attack cancer cells by presenting specific cancer‐related antigens to the immune system. Cancer‐associated antigens are often proteins that are overexpressed or specifically expressed on the surface of cancer cells. Types of cancer vaccines include: (i) dendritic cell vaccines, in which dendritic cells are harvested from the patient's blood and re‐injected into the patient after exposure to a cancer‐specific antigen; (ii) peptide vaccines, in which peptides derived from cancer‐specific proteins are administered to the patient; and (iii) DNA vaccines, in which a gene encoding a specific cancer antigen is administered directly to the patient's cells.^8^ The first cancer vaccine approved for clinical use was Sipuleucel‐T, which is used to treat advanced prostate cancer. This is a type of dendritic cell vaccine in which autologous peripheral blood mononuclear cells collected from the patient are exposed to a fusion protein consisting of prostate acid phosphatase (PAP), an antigen of prostate cancer cells, and granulocyte‐macrophage colony‐stimulating factor (GM‐CSF). Dendritic cells in the peripheral blood mononuclear cell (PBMC) isolate take up this fusion protein and present the PAP antigen to adaptive immune cells, such as T cells. Cytotoxic T lymphocytes (CTLs) that are able to recognize PAP as a foreign antigen through this process are injected back into the patient.^9^ Sipuleucel‐T has been shown to improve overall survival in some metastatic castration‐resistant prostate cancer patients. However, like several other ACT‐type therapies, can have side effects including headache, fever, chills, and even neurological complications. Additionally, ACT and cancer vaccines are expensive and survival outcomes are somewhat dismal compared to other therapies.

One of the most frequently utilized cancer immunotherapies is immune checkpoint inhibitor (ICI) therapy. ICI has emerged as a powerful tool to re‐activate the immune response to cancer and has led to major advances in cancer immunotherapy. This approach targets checkpoint proteins, specific molecules on the surface of immune cells that regulate the immune system's response to cancer cells.^10^ Immune checkpoint proteins serve as brakes that prevent excessive immune responses and the immune system from running amok against normal cells. However, cancer cells exploit these checkpoint pathways to evade immune system attacks, enabling tumor growth and progression. By blocking these checkpoint proteins, ICI therapy can help the immune system identify and attack cancer cells more effectively. Checkpoint proteins are found on the surface of immune cells, including T cells, and cancer cells, and act as important signaling molecules that stimulate (immunoactivation) or inhibit (immunosuppression) immune responses. Two important immune checkpoint proteins that have been extensively studied in oncology are programmed cell death protein 1 (PD‐1) and cytotoxic T lymphocyte‐associated protein 4 (CTLA‐4). PD‐1 is a checkpoint protein on the surface of T cells that, when interacting with its ligands PD‐L1 and PD‐L2, suppresses T‐cell activity and prevents the immune system from attacking cancer cells. This interaction allows cancer cells to successfully hide from the immune system, allowing the tumor to escape detection when it is small enough to be eliminated.^11^ CTLA‐4, another checkpoint protein found on T cells, acts earlier in the immune response than PD‐1 and regulates the initial activation of T cells; when CTLA‐4 binds to CD80 or CD86 on antigen‐presenting cells (APCs), it inhibits the immune response and prevents overactivation of the immune system.^12^, ^13^ By upregulating the expression of immune checkpoint proteins, particularly PD‐L1 secretion, cancer cells can interact with PD‐1 on T cells, effectively inhibiting the immune response to the tumor. Recognizing the importance of immune checkpoint proteins in immune evasion of cancer, researchers have developed drugs called ICIs. These drugs target and block interactions between immune checkpoint proteins such as PD‐1/PD‐L1 and CTLA‐4 to restore the ability of T cells to recognize and attack cancer cells.

ICIs have shown remarkable success in the treatment of various types of cancer, with some patients achieving long‐term responses. This was quickly followed by the development of monoclonal antibodies targeting PD‐1 (pembrolizumab and nivolumab) and PD‐L1 (atezolizumab and durvalumab). Anti‐PD‐1/PD‐L1 antibodies have since become some of the most commonly prescribed anti‐cancer therapies. T‐cell‐targeted immunomodulators are now used as standalone treatments or in combination with chemotherapy as first or second lines of treatment for about 50 types of cancer.^14^ Although ICIs have revolutionized cancer treatment, not all patients respond to these treatments. Ongoing research aims to identify biomarkers and combination therapies that could increase the effectiveness of checkpoint inhibitors and extend their utility to more cancer types.

## 3 ADVANTAGES AND LIMITATIONS OF T CELL‐TARGETED MODULATORS IN IMMUNOTHERAPY

As mentioned above T‐cell‐targeted modulators such as ICIs and CAR‐T therapies, have been predominantly utilized for cancer treatment compared to other cancer immunotherapies available. Three different ICIs, PD‐1 inhibitors, PD‐L1 inhibitors, and CTLA‐4 inhibitors, have been approved by the U.S. Food and Drug Administration (FDA) for the treatment of various cancer types.^15^ However, these ICIs still face challenges that need to be resolved. The biggest challenge is their low response rate. In recent years, ICIs have been used in a variety of cancers, but the response rate is only 20%–40%, so there is a need to develop biomarkers to predict efficacy.^16^

The terms “hot” and “cold” tumors are often used to describe the immune response to cancer: hot tumors have a strong immune response, a high number of TILs, and high immune cell activation; in contrast, cold tumors have a weak immune response, a low number of TILs, and a resultant low level of immune cell activation.^17^ ICI is most effective in hot tumors, for example, cancers that have been invaded by T cells and form inflamed tumors. Further, ICI can suppress T‐cell inhibitory interactions. Tumors exhibit extensive DNA mutations which cause the production of tumor‐characteristic neoantigens on the cell surface. These neoantigens make the tumor more visible by the immune system and trigger a strong immune response.^18^ Checkpoint inhibitors release the brakes that tumors put on T cells, allowing T cells to be more effective in killing tumors. Melanoma,^19^ kidney cancer,^20^ and non‐small cell lung cancer^21^ rely heavily on immune checkpoints for growth, and are known as hot tumors with strong immune responses, and patients with these cancers tend to have the best response to ICI therapy.

In contrast, a cold tumor is a cancer that, for various reasons, is not recognized by the immune system or does not elicit a strong response. T cells cannot penetrate such tumors and are excluded by components of the microenvironment. The tumor cells and their surrounding microenvironment are composed of blood vessels, structural elements, and specialized immune cells, including myeloid‐derived suppressor cells (MDSCs) and regulatory T cells (Tregs). Tregs secrete immunosuppressive biochemical transmitters such as cytokines that inhibit T‐cell migration into the tumor. Soft tissue cancers (such as prostate, breast, and pancreatic) most frequently present as “cold” tumors and have a very limited response to immunotherapy due to low tumor immunogenicity and an active immunosuppressive microenvironment.^22^, ^23^

MDSCs, tumor‐associated macrophages (TAMs), and tumor‐associated neutrophils (TANs) are known to be involved in tumor immune evasion. These cells create an immunosuppressive environment within the tumor, inhibiting TIL activity and preventing the immune system from attacking cancer cells.^24^ MDSCs are immature immune cells of myeloid lineage that can be recruited from circulation, spleen, and bone marrow.^25^, ^26^ These cells display surface markers shared with neutrophils and monocytes, possess potent immunosuppressive activity, and play an important role in regulating immune responses in a variety of pathological conditions, including chronic infections and autoimmune diseases as well as cancer.

In general, two major subsets of MDSCs have been distinguished: monocytic MDSCs (mMDSCs, M‐MDSCs) and granulocytic/polymorphonuclear MDSCs (gMDSCs, also abbreviated as G‐MDSCs or PMN‐MDSCs).^25^, ^26^ Granulocytic MDSCs, also called neutrophilic MDSCs, share many features with normal neutrophils but exhibit additional immunosuppressive functions. MDSCs suppress T‐cell activity and proliferation by producing high levels of arginase‐1 (Arg1) and inducible nitric oxide synthase (iNOS) and reactive oxygen species (ROS).^27^ In addition, MDSCs secrete immunosuppressive cytokines such as interleukin 10 (IL‐10) and transforming growth factor‐β (TGF‐β), which inhibit immune cell activation and proliferation.^28^ MDSCs also attract Tregs to the TME, further weakening the anti‐tumor immune response.

The regulation of suppressive myeloid cell is important to increase the effectiveness of ICI therapy for cold tumor. Even though MDSCs have been studied for years, their origin and development remain largely unclear. A consensus among scientists exists related to the development of MDSCs from myeloid cells that are defined by their immunosuppressive function and myeloid origin, but do not represent a well‐defined, single‐cell subset. Additionally, no cell surface markers specific to MDSCs have been identified which further complicates targeting of MDSCs.^29^, ^30^

There is a lot of evidence that MDSCs promote the “cold” environment of soft tissue tumors; however, there aree additional supportive data that tumor‐infiltrating MDSCs may, in fact, be suppressive monocytes or neutrophils. Based on the abundance of data supporting this, it would seem that TMEs that have poor T‐cell infiltration along with abundant numbers of innate immune cells, like neutrophils, would respond better to more myeloid, specifically neutrophil‐focused therapy. In this review, we focus primarily on neutrophils because of the increasing amount of supportive evidence demonstrating their regulation of several cancer types.

## 4 NEUTROPHILS AND THEIR ROLES IN THE TME

Neutrophils, one of the most abundant immune cells in the body, have been recognized for their role in combating infections and pathogens. Their highly potent anti‐microbial properties have been well characterized and in recent years, their contribution to cancer development and progression has been drawing attention. However, with regard to their role in cancer, there are several viewpoints on whether neutrophils are pro‐ or anti‐tumorigenic, leaving a lot to be discovered and leveraged for anti‐cancer therapy. Neutrophils have been shown to promote tumor growth and metastasis both directly and indirectly through various mechanisms that include the secretion of growth factors and specific neutrophil granular enzymes as well as crosstalk with other epithelial cells and immune cells, ultimately facilitating cancer invasion, metastasis, tumor progression and suppression of the anti‐tumor immune response.^31^, ^32^

Neutrophils have been shown to function at multiple tissue sites and within several stages of cancer progression: at the primary tumor site, within lymph nodes and secondary hematopoietic organs, and at secondary tissue sites of metastasis. For example, a previous study showed that neutrophils are recruited from bone marrow to 4T1 mammary tumors, where they promote mammary tumor progression.^33^ This finding was further supported in a separate study demonstrating the impact of neutrophils on 4T1 lung metastasis through their function in lung tissue.^34^ Whereas, Wang et al. recently demonstrated neutrophils to be recruited into spleens of 4T1 tumor‐bearing mice, where they actively suppress T‐cell activation and anti‐tumor immune responses.^35^ These interesting findings highlight the diversity of mechanisms (and in multiple tissue microenvironments) that neutrophils can utilize to regulate tumor progression.

Neutrophils are also receiving greater attention due to their potential as biomarkers in many different cancer types. A high neutrophil‐to‐lymphocyte ratio (NLR) is used to indicate prognosis in a variety of cancers, though not with controversy considering the correlation of high NLR with both poor and good prognosis depending on treatment regimen and cancer type.^36^, ^37^, ^38^ The role of neutrophils in cancer is highly complex, hence providing multiple avenues for determining how precisely their diverse functions contribute to tumor growth. Understanding neutrophil function can offer novel therapeutic approaches for cancer treatment.

This review aims to emphasize the potential that neutrophils hold promise for, thus opening the door for cancer immunotherapeutic developments beyond T‐cell approaches such as checkpoint inhibition, more relevant to cancers with an abundance of T‐cell infiltration compared to other “cold” tumors or cancer microenvironments with less T‐cell representation.

## 5 THE UNIQUE FEATURES OF NEUTROPHILS

Neutrophils are crucial components of the innate immune system, playing a vital role as the body's first line of defense against invading pathogens.^39^ They are classified as granulocytes as they possess cytoplasmic granules and demonstrate a range of unique approaches to combating pathogens, including phagocytosis, where they engulf and destroy foreign cells, immune complexes, and viruses. Additionally, neutrophils are key drivers of inflammation through strong cytokine/chemokine signaling, exhibit chemotaxis and transmigration, undergo degranulation, produce ROS, and form extracellular traps, mechanisms that collectively induce microbial cell death.^40^ Notably, neutrophils also contribute to wound healing processes, which is pertinent to their function in tumors, which has been classically described as a “wound that won't heal”.^41^, ^42^

**FIGURE 1:**
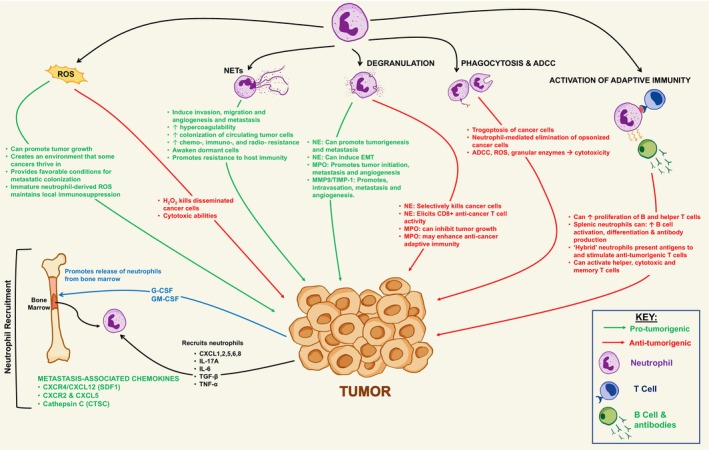
Roles of neutrophils in the tumor microenvironment (TME). A comprehensive overview of the intricate relationship between neutrophils and cancer cells in the TME. This figure shows the process of neutrophil recruitment and highlights their vital functions in the TME which may be both pro‐ or anti‐tumorigenic, including the production of reactive oxygen species (ROS), neutrophil extracellular traps (NETs), degranulation, phagocytosis, mediating antibody‐dependent cellular cytotoxicity (ADCC), and the emerging roles of neutrophils in the activation of adaptive immunity.

In addition to predominant mediators of innate immunity, neutrophils also play a significant part in adaptive immunity and foster crosstalk with various immune cells. Their production of numerous cytokines and chemokines enables interaction with a diverse range of immune cells including endothelial cells, dendritic cells, macrophages, natural killer cells, T lymphocytes, and B lymphocytes.^39^ In fact, emerging research finds the previously unrecognized ability of neutrophils to engage in antigen presentation, which contradicts the conventional idea that neutrophils do not possess the ability to present antigens to T cells. This will be discussed in more detail in subsequent sections of this review. These aspects highlight the importance of neutrophils in bridging innate and adaptive immunity, and their unique and distinct properties render them highly effective in immune defense (Figure 1).

Other granulocytes include basophils and eosinophils, which have been established as crucial players in the host defense against parasites and inflammation associated with allergic responses. Basophils have been shown to be present in the landscape of tumors where they contribute to angiogenesis, tumor progression and display unique properties in cancer. However, more in‐depth studies are required for a complete understanding of the roles they play in human cancer and to elucidate whether they are beneficial, harmful, or neutral in cancer.^43^, ^44^ In recent years, eosinophils have come into focus with studies showcasing their potential roles in cancer. Eosinophils have been recognized for complex roles (both pro‐ and anti‐tumorigenic) that encompass their cytotoxic capabilities and secretion of enzymes, cytokines, chemokines, and angiogenic factors. Furthermore, some research highlights indirect anti‐tumor mechanisms through T‐cell activation or macrophage polarization.^45^, ^46^ However, cancer studies on basophils and eosinophils have been relatively limited, compared to neutrophils. While neutrophils have been extensively researched for their roles in cancer progression in recent years, the functions of the other granulocytes remain less understood and require further investigation. This article will thus explore the multifaceted roles of neutrophils and the future potential that they hold in the context of cancer.

### 5.1 Neutrophil recruitment

Neutrophils recruited via chemokines to sites of inflammation or infection exhibit cell migration strategies including tethering, rolling, adhesion, crawling, and transmigration. This occurs via activated endothelial cells which express cell adhesion molecules, that bind to and allow migration of neutrophils via integrin interactions.^47^, ^48^ In a similar fashion, neutrophils are often recruited to the site of tumors by chemokines produced in the TME. This recruitment is predominantly via CXCR1 and CXCR2 receptors shown by reduced migration of neutrophils upon the inhibition of these receptors on the neutrophils. Common chemokines that are being established to be potent neutrophil chemo‐attractants are CXCL1, 2, 5, 6, and 8 which are reviewed to be upregulated across several cancer types, and to play crucial roles in the infiltration by TANs.^49^, ^50^, ^51^, ^52^, ^53^ A common occurrence appears to be the recruitment of neutrophils by TME‐derived IL‐8 (CXCL8)^52^, ^54^, ^55^ which has been shown to be upregulated in various cancer types, and not only does it contribute to neutrophil recruitment, but has also been shown to be pro‐tumorigenic by promoting angiogenesis, stemness of cancer cells and the epithelial‐to‐mesenchymal transition (EMT) of cancer cells.^54^ There have also been reports that several interleukins namely IL‐17A and IL‐6 as well as other cytokines, such as tumor necrosis factor‐alpha (TNF‐α) and TGF‐β contribute to neutrophil recruitment and the regulation of their function.^53^, ^56^, ^57^, ^58^, ^59^ In fact, TGF‐β has been found to also regulate neutrophil plasticity as identified by Fridlender et al.^57^ This crucial immunosuppressive modulator is usually overexpressed in tumor cells and drives neutrophils to a pro‐tumorigenic phenotype. In addition to these cytokines and chemokines, granulocyte‐colony‐stimulating factor and GM‐CSF are critical growth factors often upregulated in cancer, which promote the release of mature neutrophils out of the bone marrow and into the systemic blood stream^58^ which in turn increases the survival of neutrophils.

It is also worth noting the importance of these factors in the recruitment of TANs toward pre‐metastatic niches. It has been well established that the neutrophil chemokine axis CXCR4/CXCL12 is crucial in recruiting neutrophils to the pre‐metastatic niche such as in breast cancer,^60^ and CXCL12 is upregulated in several different metastatic sites as reviewed by Chen and Yu.^61^ This review reported illustrates the importance of the CXCR2‐dependent recruitment of neutrophils and the role of CXCL5 in promoting both recruitment of neutrophils to lymph nodes as well as lymph node metastasis. Furthermore, the stromal cell‐derived factor 1 (SDF1/CXCL12)‐CXCR4 axis also contributes to neutrophil recruitment to the liver pre‐metastatic niche.^62^ Neutrophils recruited to the site of metastasis have been shown to promote tumor progression such as a study that showed that the tumor‐secreted protease cathepsin C (CTSC) promotes metastasis from breast to the lung by enhancing the infiltration of neutrophils and the formation of neutrophil extracellular traps (NETs), which in turn promotes tumor cell extravasation and their colonization in the lungs.^56^

On the contrary, the presence of neutrophils in the pre‐metastatic niche does not always have negative implications. A study demonstrated that proto‐oncogene c‐met (MET), induced by tumor‐derived TNF‐α among other inflammatory stimuli, promotes neutrophil attraction to both primary and metastatic sites. Furthermore, MET stimulates the production of iNOS, leading release of nitric oxide by neutrophils which promotes cancer cell killing and thus reduces tumor growth and metastasis.^63^ Additionally, it has been demonstrated that CCR2‐dependant tumor entrained neutrophils present in the pre‐metastatic lung, can inhibit the seeding of tumor cells and suppress the colonization of tumor cells in the lung.^64^ This highlights that while tumor‐secreted factors may aid in tumor growth at primary sites, in parallel, they induce a neutrophil‐mediated inhibitory mechanism at metastatic sites.

## 6 NEUTROPHIL BACTERICIDAL MECHANISMS AND TUMOR PROGRESSION

Upon recruitment into the TME, neutrophils modulate tumor progression through a number of bactericidal mechanisms, including ROS secretion, degranulation/secretion of granule enzymes, phagocytosis, and antibody‐dependent cellular cytotoxicity (ADCC). Although these classical properties target pathogens and are focused on the resolution of infections, these same tools can modulate cancer disease progression in a very context‐specific manner.

### 6.1 Neutrophil‐derived ROS

The precise role of neutrophil‐derived ROS in cancer remains incomplete, with differing perspectives on their complex functions and potential implications. A study investigating the cytotoxic abilities of neutrophils against cancer^65^ found that catalase, responsible for the decomposition of hydrogen peroxide (H_2_O_2_), reduced neutrophil‐induced lung cancer cell killing which suggests that neutrophil‐derived H_2_O_2_ produced is required for this immune response. However, the inhibition of NADPH oxidase did not significantly alter neutrophil‐mediated lung cancer death and instead increased their cytotoxicity, indicating that ROS production by neutrophils is not required for the anti‐cancer outcome they had observed. Similarly, a recent study from our group showed that neutrophil ROS depletion via genetic deletion of NADPH oxidase deletion enhanced neutrophil cytotoxicity against subtypes of bone metastatic prostate cancer. We had previously noted that neutrophils undergo oxidative burst (release ROS) when in contact with prostate cancer cells^66^ and respond to soluble cancer factors in conditioned media. However, various methods of ROS depletion suppressed prostate tumor growth in the bone and additionally, it was found that metastatic prostate cancer induces pathways associated with oxidative stress response in neutrophils. The prostate cancer cells can resist neutrophil‐mediated death through cancer alterations in glutathione synthesis, a dominant cellular antioxidant. These findings highlight that some cancers thrive under neutrophil‐derived oxidative stress conditions, yet still show promise in targeting ROS and glutathione programming.^67^

Granot et al. reported that tumor‐entrained neutrophils accumulate in the pre‐metastatic lung of breast tumor‐bearing mice and provide protection against metastasis by killing disseminated tumor cells. The group identified that these entrained neutrophils inhibit the seeding of disseminated cells in the lung by killing the cancer cells in an H_2_O_2_‐dependent manner,^64^ and that the cytotoxic ability of neutrophils is mediated through the NADPH oxidase–H_2_O_2_ pathway triggered by the secretion of H_2_O_2_ in neutrophils upon physical contact between cancer cells and neutrophils. Building upon these findings, they demonstrated that H_2_O_2_ induces a lethal influx of Ca^2+^ in tumor cells, facilitated by the transient receptor potential cation channel, subfamily M, member 2 (TRPM2). TRPM2, known to be H_2_O_2_‐dependent and frequently upregulated in cancer renders tumor cells more vulnerable to neutrophil‐mediated killing, and cells expressing reduced levels of TRPM2 were protected from neutrophil cytotoxicity and exhibited enhanced seeding in the pre‐metastatic lung.^68^

Conversely, in some contexts, neutrophil‐released ROS can promote favorable conditions for tumor progression. Zhong et al. Using a natural mutation in the *Ncf1* gene (encoding a cytosolic component of the NADPH oxidase 2 [NOX2] complex), a group identified that *Ncf1* competent neutrophils exhibited functional induction of ROS and IL‐1β signaling, thus promoting lung colonization by tumor cells.^69^ Another group, Rice et al., revealed that a specific subset of immature neutrophils (c‐Kit+ neutrophils) can engage in oxidative mitochondrial metabolism, as opposed to traditional neutrophils which are primarily glycolytic. c‐Kit+ neutrophils use mitochondrial fatty acid oxidation to produce ROS when glucose availability is limited in the mammary TME. Further, tumor‐induced oxidative neutrophils showed sustained ROS production and T‐cell suppression, suggesting that these metabolically adapted, oxidative neutrophils within the glucose‐restricted TME play a crucial role in maintaining local immune suppression.^70^

### 6.2 NETosis

As previously described, NETosis is a unique neutrophil function and is the process of generating and extruding NETs (filamentous extracellular structures made of modified chromatin decorated with neutrophil anti‐microbial peptides and citrullinated histones) in response to pathogens.^71^ NETs have emerged as an important point of interest in cancer research due to their significant involvement in several cancer‐related processes. NET formation can be induced by pathogens and their pathogen‐associated molecular patterns such as lipopolysaccharides, antibodies, ROS, cytokines such as IL‐8 and TNF, chemokines including CXCR1 and CXCR2 agonists, and physiological stimuli.^72^, ^73^, ^74^ In fact, a recent study showed how Cathepsin C can promote lung cancer metastasis of breast cancer cells by inducing neutrophil infiltration and NETosis.^56^

Numerous reports highlight the detrimental role of NETs in promoting tumor progression and metastasis, including stimulation of cancer cell migration, invasion, and angiogenesis as well as hypercoagulability by inducing plasma thrombin generation.^75^, ^76^ For example, an investigation showed that plasma redox imbalance, due to albumin oxidation, promotes NETosis which contributes to the colonization of circulating tumor cells in the lung, thus promoting pulmonary metastases.^77^ Along those lines, a separate study revealed that NETs sequester circulating lung carcinoma cells and rapidly promote colonization and metastasis.^78^ Metastatic breast cancer has also been shown to induce neutrophils to form metastasis‐supporting NETs to facilitate the dissemination of breast cancer cells to the lungs in mice.^34^ NETs have also been shown to assist in the development of a pre‐metastatic niche. Specifically, Lee et al. observed that ovarian tumors stimulate NETosis, and notably, NETs were detected in the omentum prior to metastasis in both tumor‐bearing mice with tumors and early‐stage ovarian cancer patients, suggesting their critical role in promoting ovarian tumor metastasis to the omentum.^79^

Other note‐worthy investigations provide evidence that NETs promote liver metastases after surgical stress,^80^ and NETs in pre‐metastatic livers promote cancer metastasis of breast and colon cancers, which were attributed to NET interactions with the transmembrane protein CCDC25 on tumor cells, which enhances cell motility.^81^ NETs were also reported to awaken dormant tumor cells in mice in pre‐metastatic niches by Albrengues et al., wherein lung inflammation induced the formation of NETs which contributed significantly to awakening dormant cancer cells. Particularly, NET‐associated neutrophil elastase (NE) and matrix metalloprotease 9 (MMP‐9) cleaved laminin which triggered the proliferation of dormant cancer cells.^82^

In addition to tumor‐regulatory mechanisms, NETs have been shown to be instrumental in acquired resistance to chemotherapy and immunotherapy.^83^ For instance, NETs contributed to resistance to doxorubicin and melphalan in a mouse model of multiple myeloma.^84^ A landmark study conducted by Mousset et al. illustrated that chemotherapy can trigger NET formation, which induces treatment resistance via TGF‐β activation.^85^ The study explores how chemotherapy upregulates CXCL1 and CXCL5, in cancer cells in ling metastases, resulting in neutrophil recruitment. Furthermore, chemotherapy‐treated tumor cells which are dying release active IL‐1β, provoking the recruited neutrophils to form NETs in lung metastases. NET‐associated proteins, integrin‐αvβ1, and MMP‐9, synergistically capture and activate latent TGF‐β, which interacts with the TGFβR1 on cancer cells and promotes EMT as well as chemoresistance. NETs have also been documented to have an impact on radiation resistance wherein increased NETs in the TME were observed post‐radiation in a bladder cancer model. NET blockade enhanced the radiation response, and interestingly, NETs were detected in bladder tumors of non‐responsive patients post‐radiation, thus implying the involvement of NETs in radiation resistance.^86^

It is important to also note, that NETs have been indicated to also promote resistance to the host's adaptive anti‐cancer response. Teijeira et al. demonstrate how NETs shield cancer cells from cytotoxic T cells and NK cells. NETs wrap around tumor cells, impeding their interaction with cytotoxic immune cells, and this process in turn facilitates metastases in mice. This emphasizes the importance of recognizing how NETs can hinder effective anti‐tumor immune responses and contribute to immune evasion.^87^ These remarkable studies offer promising avenues for targeting NETs, which can help hinder several aspects of tumor progression.

### 6.3 Degranulation

Degranulation is the process by which neutrophils release their various unique peptides from the granules stored in their cytoplasm. This process occurs either at the plasma membrane, thus releasing these peptides out of the neutrophil to kill extracellular microbes, or into the phagosome to concentrate these peptides in the phagosome to kill intracellular microbes.^88^ Neutrophils have four classes of granules: (i) primary, or azurophilic, granules which store highly toxic peptides such as NE, myeloperoxidase (MPO), cathepsins, and defensins, (ii) secondary, or specific, granules which contain lactoferrin and other substances, (iii) tertiary granules containing cathepsins, MMP‐9/gelatinase B and MMP‐8, among other substances,^88^, ^89^ and (iv) secretory vesicles, primarily known to store albumin, which are formed by endocytosis.^88^, ^90^ Neutrophil granules cannot be liberated until the cytoplasm receives a signal from receptors in the plasma or phagosomal membrane. This is a tightly controlled process as neutrophils are rich in tissue‐destructive proteases. These molecules aid in the breakdown and neutralization of engulfed substances as well as extracellular matter, facilitating their destruction, and play crucial roles in other processes. However, these proteases have also been observed to have diverse roles in cancer, but their impact has not conclusively been characterized as either beneficial or detrimental.

MMP‐9, a component of the tertiary granules, is shown to be secreted by neutrophils in the TME of multiple cancer types. In cancer, neutrophil‐derived MMP‐9 has been gaining attention for its role in promoting metastasis and angiogenesis. Ardi et al. demonstrated that human infiltrating neutrophils release a unique Tissue Inhibitor of Metalloproteinase (TIMP)‐free MMP‐9 which strongly stimulates angiogenesis. proMMP‐9 alone induced angiogenesis at subnanogram levels while neutrophil proMMP‐9 with TIMP‐1 failed to induce angiogenesis; this was verified by both synthetic and natural forms of proMMP‐9/TIMP‐1 complexes derived from human cells.^91^ A recent investigation conducted by this team revealed that tissue‐infiltrating neutrophils are the primary source of in vivo angiogenesis‐inducing MMP‐9 in the TME. TANs surpass the levels of MMP‐9 expression secreted by TAMs and trigger more efficient in vivo angiogenesis and a faster response. By significantly influencing the microarchitecture of angiogenic vessels, these neutrophils ultimately facilitate the dissemination of tumor cells.^92^ Neutrophil‐derived TIMP‐free MMP‐9 has also demonstrated the ability to facilitate the intravasation of malignant cells.^93^

Another primary granule enzyme, NE has been a recipient of attention regarding its pro‐tumorigenic roles, as NE is suspected to promote tumor progression and metastasis of breast cancer.^94^, ^95^ These reports reveal that NE in cancer tissue can be an independent prognostic factor for breast cancer demonstrated by poor survival. NE inhibition has also been shown to suppress tumor growth as well as the development of metastatic foci.^94^, ^96^ NE has also been shown to promote inflammation‐mediated lung cancer via the activation of the IL‐8/CXCR2 pathway which in turn recruits neutrophils that release NE.^97^ NE expression and activity is said to be upregulated in several cancer types and mouse models of cancer,^98^ for example, human colorectal cancer xenografts possess very high NE activity.^99^ As reviewed by Lerman and Hammes, NE can directly increase the release of VEGF from tumor cells, thereby activating endothelial proliferation. They also suggest that NE facilitates metastasis by inducing EMT, resulting in more invasive cancer cells.^98^ In support of this, an investigation studying Lewis lung carcinoma in *Elane* ^−/−^ (the gene encoding NE) mice showed significantly slower tumor growth as well as a reduction in number and size of metastatic foci. The group also demonstrated that NE activates the phosphoinositide 3‐kinase‐Akt proliferative pathway by the internalization and degradation of insulin receptor substrate 1 in lung cancer cells.^100^ Another study involving lung cancer revealed that NE degrades thrombospondin‐1, an anti‐tumorigenic factor, thus increasing the growth of lung tumors and metastatic melanoma foci.^101^ However, Cui et al. made an intriguing discovery demonstrating that human NE (or, ELANE) has the ability to selectively kill a variety of cancer cells while sparing neighboring non‐cancer cells.^102^ Moreover, the group revealed that ELANE effectively reduced primary tumor growth and elicited a CD8^+^ T cell‐mediated abscopal effect against distant metastases. This innovative discovery holds promise for the development and optimization of anti‐cancer therapy^103^ but demonstrates the complexity and diversity of NE function and contribution to tumor progression.

MPO is critical in catalyzing the conversion of H_2_O_2_ to forms of ROS having potent anti‐microbial activity,^104^ and these neutrophilic enzymes have been shown to be very powerful in the proteolytic processing and thus activation of molecules and interleukins such as IL‐1, IL‐18, and IL‐33, which trigger several downstream inflammatory pathways and communication.^105^, ^106^ MPO has been associated with tumor initiation by facilitating a hypermutagenic environment (from MPO‐derived oxidants that oxidize and modify DNA) as reviewed by Valadez‐Cosmes et al.^107^ It is also believed that MPO influences tumor growth, apoptosis, and metastasis. By inhibiting MPO, groups have observed reduced tumor burden in lung cancer models, as well as a reduction of tumor size in a tumor graft model in MPO‐knockout mice using Lewis lung carcinoma cells.^107^ Furthermore, MPO treatment has also been shown to enhance primary breast tumor growth and their lung metastasis.^108^ In this study, MPO has been shown to recruit breast cancer cells and induce the transcription of MMP1, MMP3, and COX‐2 genes (pro‐tumorigenic and organ‐specific metastatic genes) and augment pro‐tumorigenic collagen production and angiogenesis.

It is intriguing to discover that MPO and the hypochlorous acid (HOCl) it produces are involved in adaptive immunity, which is unexpected. Neutrophil‐derived HOCl has been shown to oxidize proteins, thereby increasing their immunogenicity which may enhance adaptive immunity.^108^ HOCl has been proposed in this review to act as an adjuvant to boost adaptive immune responses, supported by studies showing that dendritic cells process and present HOCl‐modified ovalbumin more effectively to CD4 and CD8 T‐cells than native ovalbumin. A separate study showed that inhibiting MPO improved outcomes of immune checkpoint therapy for melanoma, as ROS from immunosuppressive myeloid cells enable an immunosuppressive TME.^109^ While a growing body of evidence highlights the involvement of granular enzymes in cancer, a significant knowledge gap still remains in completely understanding the vast roles of other granular enzymes. These insights will open up a potential route for anti‐cancer therapy.

### 6.4 Phagocytosis and ADCC

Phagocytosis, one of the primary mechanisms of destruction of microbes by neutrophils, involves the engulfment of the microorganism into a phagocytic vacuole which matures to form a phagososome where the microorganism is eliminated by low pH, and potent anti‐microbial enzymes.^47^, ^110^ Not only is this process important for killing microbes but is also effective in tissue homeostasis for the clearance of dead cells and tissue debris.^111^ Neutrophils are capable of efficiently performing phagocytosis and ADCC against microbial cells. However, due to their relatively smaller size compared to cancer cells, it is uncommon for neutrophils to completely engulf cancer cells. Instead, they engage in a distinct process called trogocytosis or trogoptosis, where they nibble or take “bites” off the cancer cell membrane, which results in the loss of cell membrane integrity and thus, cell death.^112^, ^113^ Neutrophil‐mediated elimination is most efficient when the target cancer cells are opsonized with therapeutic antibodies via antibody‐dependent cellular phagocytosis (ADCP). Moreover, the direct cytotoxicity by neutrophils has also been reported to be mediated by the release of ROS and cytotoxic granular components upon degranulation. Neutrophils express various Fc receptors, which can be activating or inhibitory, to respectively activate or suppress ADCP, ADCC, and trogocytosis.^112^ The therapeutic targeting of cancer cells, utilizing approaches such as HER‐2/neu‐directed monoclonal antibodies or FcR‐directed bispecific antibodies,^114^ has demonstrated how neutrophils participate in the elimination of cancer cells through ADCC. Multiple studies and reviews^113^, ^115^ have provided evidence supporting the role of neutrophils in abolishing cancer cells by ADCC and leading to a lytic, or necrotic type of cancer cell death when engaged by these targeted therapeutic agents.

## 7 ACTIVATION OF ADAPTIVE IMMUNITY

Traditionally, neutrophils have been regarded as short‐lived, innate immune cells that are not involved in antigen presentation. However, recent reports have challenged this concept indicating that neutrophils can present antigens, acting as APCs and as cells with T helper‐like functions. Specifically, neutrophils were shown to migrate from sites of bacterial infection to lymph nodes where they mediate the proliferation of both B cells and helper T cells; inhibition of neutrophil migration resulted in a reduction in T‐cell proliferation.^116^ This landmark study brought out a fascinating connection between innate immunity (neutrophils) and adaptive immunity (T and B cells) but did not ascertain whether neutrophils actually act as APCs. Mysore et al. have reported recently that murine neutrophils endocytose antigen–antibody complexes via Fc receptors, get converted to APC with dendritic cell‐like properties, activate specific T helper cells similar to classical dendritic cells, and elicit cytotoxic T‐cell activity against tumors.^117^ They also demonstrated that human neutrophil‐derived APC activate antigen‐specific memory T cells in vitro, leading them to propose the exciting suggestion that neutrophil‐APCs could serve as potent activators of T cells for immunotherapy.

Additionally, the work of Puga et al. led to the identification of a subpopulation of neutrophils in the marginal (B cell‐rich) zone of spleens with the ability to stimulate antibody production by B cells in a cytokine‐mediated process.^118^ This study on mice, monkeys, and humans showed that co‐culture of splenic neutrophils with B cells results in the activation and differentiation of B cells as well as class switching. In other words, these splenic neutrophils behave like conventional T helper cells; interestingly, it appears that neutrophils in the peripheral blood do not have this B cell‐stimulating activity, suggesting that the splenic neutrophils are a specialized population.

In 2016, Singhal et al. reported the identification of a very interesting subset of TANs; these cells exhibit the phenotype of *both* neutrophils and APC. These cells, named “hybrid neutrophils” by these researchers, are able to present antigens and even stimulated T‐cell immunity against tumors.^119^ In fact, TANs have been shown to migrate to the lymph nodes during the early stages of progression of head and neck cancer, where they become capable of antigen presentation (with expression of HLA‐DR and other markers of classical APC) and stimulate T‐cell responses.^120^ This elegant study demonstrated that neutrophils in tumor‐draining lymph nodes acquire an APC phenotype, are able to internalize, process, and present them, and form synapses with T cells and activate them. However, during later stages of metastatic disease, the metastatic TME induced the emergence immunosuppressive neutrophils which inhibit T‐cell responses. The accumulation of neutrophils in the lymph nodes in the metastasis‐free stages of cancer was associated with a positive prognostic value for patient survival. This study suggests that neutrophils with antigen‐presenting function can have a positive impact in terms of beneficial anti‐tumor immune responses.

Neutrophils continue to surprise us with new findings on their multipotent abilities. While the studies mentioned above describe the ability of neutrophils to activate T cells, a recent study describes the so‐called “B‐helper neutrophils” in the B cell follicles of draining lymph nodes of patients with head‐and‐neck cancer. Pylaeva et al. reported that a population of neutrophils in these lymph nodes express a phenotype similar to that of T helper cells and mediate the activation and proliferation of B cells,^121^ the way classical T helper cells do. They also showed an association between high numbers of neutrophils in the regional lymph nodes and improved survival of patients with head‐and‐neck cancer. This has very significant implications, particularly in the context of cancer research, as it opens paths to the possibility that neutrophils might play critical roles not only in innate immunity but also in influencing adaptive immune responses.

In summary, neutrophils are extremely versatile and vital cells of the immune system which exhibit a wide range of abilities now being recognized as powerful players in different aspects of tumor progression and tumor suppression. These emerging roles shed light on the novel prospects in cancer research, allowing us to unlock opportunities to leverage neutrophils in the context of cancer and introduce innovative immunotherapeutic strategies.

## 8 POTENTIAL IMMUNOTHERAPEUTIC APPROACHES USING NEUTROPHILS

Researchers have attempted to target different cellular and molecular components of the TME.^122^ These components include (i) tumor‐infiltrating T cells with the objective of boosting their activation,^123^ (ii) cancer‐associated fibroblasts which have been shown to be pro‐tumorigenic,^124^ (iii) TAMs which are also known to promote tumor growth and progression,^125^ (v) immunosuppressive cytokines such as TGFβ,^126^ and (vi) Treg cells, which are typically associated with poor prognosis.^127^ Of the various components worth targeting, neutrophils deserve much attention given their impact on tumor proliferation, metastatic spreading, and abundance in the microenvironment of multiple tumor types. TANs have been shown to have tumor‐supportive capabilities such as (i) promoting tumor invasion,^128^ (ii) remodeling the extracellular matrix, (iii) secreting immunosuppressive cytokines and growth factors such as TGF‐β and hepatocyte growth factor, respectively.^128^, ^129^ Additionally, peripheral blood neutrophils are also reported to support tumor progression and metastasis of circulating tumor cells by enabling cell cycle progression.^130^ Thus, there is more than adequate justification to explore ways of therapeutic targeting neutrophils in the TME.

Because of the inherent properties of heterogeneity and plasticity in the TME, neutrophils have seemingly conflicting pro‐tumor and anti‐tumor effects. Literature is replete with studies that show that neutrophils can kill tumor cells directly and via ADCC, and studies that demonstrate that neutrophils promote tumorigenesis, metastasis, and also suppress immune cells in the TME. Indeed, cancers seem to exploit neutrophils for their own survival and growth. Thus, much research attention has been focused on ascertaining whether TANs can be manipulated by promoting their anti‐tumor activities and/or inhibiting their pro‐tumor potential. We have collectively described novel neutrophil‐targeted cancer immunotherapies below (summarized in Table 1).

**TABLE 1:** Neutrophil‐focused immunotherapy approaches.

Immunotherapeutic approaches using neutrophils	Targeted molecules	Mechanisms	Targeted tumor type(s)
Neutrophil‐activation therapy	TNF‐α, CD40	TNF‐mediated recruitment and activation of neutrophils; expansion of neutrophils and enhanced ADCC^131^	Multiple
CXCR2 inhibition	CXCR2	Improved T‐cell entry into tumor^132^; suppression of inflammation‐driven tumorgenesis^133^	Pancreatic ductal adenocarcinoma
Targeting of immunosuppressive neutrophils	CD33	Depletion of MDSCs^134^	Multi cancer types
	Up‐regulation of glutathione synthase using ATRA	Differentiation of MDSCs into mature myeloid cells^135^	Colon carcinoma; lymphoma
	PDE5	Inhibition of immunosuppressive activity of MDSCs^136^	Hepatocellular carcinoma
	Histone deacetylase inhibitor	Decreasing the frequency of circulating MDSCs via CD40^137^	Breast cancer
	COX‐2	Reduced systemic prostaglandin E2 and CCL2‐mediated accumulation of granulocytic MDSCs^138^	Glioma
	ARG1	Reverse the inhibition of T cells by blocking l‐arginine depletion^139^	Multiple
	STAT3	Blockade of MDSCs accumulation^140^, ^141^	Acute myeloid leukemia; myelodysplastic syndromes; lymphoma and non‐small cell lung cancer
	VEGF	Inhibiting a promoter for MDSCs expansion^142^	Renal cell carcinoma
	CXCR2	Rescue MDSCs trafficking and enhance anti‐PD‐1 efficacy^143^	Rhabdomyosarcoma
	CXCR4	Synergize with anti‐PD‐1 therapy^144^	Multiple
		Decreased the number of MDSCs^145^	Pancreatic cancer
CAR‐neutrophils	CARs	Engineering neutrophils to express zeta‐ and gamma‐chains of T‐cell receptors^146^	
		Genetically engineered human pluripotent stem cells to generate glioblastoma‐specific CAR‐neutrophils^147^	Glioblastoma

Abbreviations: CAR, chimeric antigen receptor; MDSC, myeloid‐derived suppressor cell; TNF‐α, tumor necrosis factor‐alpha.

### 8.1 Neutrophil‐activation therapy

Attempts have been made to activate neutrophils with the objective of getting them to infiltrate and attack solid tumors and also reduce metastasis. A recent landmark study on mouse models by Linde et al. focused on stimulating neutrophils with TNF‐α and anti‐CD40 antibody and examining anti‐tumor killing by ADCC via tumor‐binding antibodies.^131^ This three‐component treatment, which the authors termed “neutrophil‐activating therapy”, resulted in (i) TNF‐mediated recruitment activation of neutrophils both in vivo and in vitro, (ii) expansion of neutrophils and enhanced cytotoxicity by anti‐CD40 antibody and (iii) lysis of human tumor cells (in vitro) and tumor clearance (in vivo) by ADCC. Linde et al. also described the mechanism of action of this therapeutic approach; complement activation stimulated the production of ROS which caused oxidative damage of tumor cells. This promising approach suggests that neutrophils can indeed be harnessed for potential anti‐tumor activity. The authors argue that the plastic nature of neutrophils can be exploited, by stimulating them rather than by inhibiting suppressive neutrophils. Linde et al. opine that this neutrophil‐activating approach induces an inflammatory cascade that could be used against “cold” tumors in which infiltration by T cells is poor or ineffective.^131^

### 8.2 CXCR2 inhibition

CXCR2 is a receptor for human chemokines CXCL1, CXCL2, CXCL3, CXCL5, CXCL6, CXCL7, and CXCL8 (IL‐8), and is a major controller of neutrophil migration from the bone marrow. More pertinently, CXCR2 promotes the recruitment of neutrophils to sites of inflammation. In the context of cancer, CXCR2 has been implicated in the tumorigenesis of skin and colon cancer,^133^ and more recently, has been implicated in tumor metastasis.^148^ Steele et al.^132^ showed that CXCR2 expression is associated with poor outcomes in patients with human pancreatic ductal adenocarcinoma (PDAC). Further, systemic genetic deletion of CXCR2 and/or antibody‐mediated depletion of neutrophils inhibited metastasis and prolonged survival in a mouse model of PDAC. These data suggest that inhibition of CXCR2 would be of benefit in human cancer for preventing metastasis and enhancing the efficacy of immunotherapeutic regimens.

Several other interesting approaches have been suggested, but these need more intensive investigation. For example, Blaisdell et al. showed that neutrophils hinder tumor growth and slow down malignant progression by causing the detachment of tumor cells from the basement membranes in uterine cancer in mice; interestingly, this process does not require contributions from other leukocytes.^149^

### 8.3 Targeting of immunosuppressive neutrophils

There is a strong justification for focusing on the immunosuppressive nature of neutrophils or immunosuppressive neutrophil phenotypes to induce anti‐tumor immune actions by other immune cells including T cells, macrophages, NK cells, and even neutrophils, themselves. It is now generally accepted that chronic inflammation (i.e., unresolved inflammation) aids and abets tumor growth and spread^150^; Souto et al. suggested more than a decade ago that a shift to acute inflammation may convert neutrophils into potent anti‐tumor effectors.^151^ They proposed an “intense and sustained neutrophilia as a treatment against solid tumors”.

In addition to activating anti‐tumor neutrophil activity, a mutually non‐exclusive strategy would be to selectively target neutrophils with immunosuppressive activity. Solid tumors have been shown to be infiltrated by MDSCs which are described as “pathologically activated neutrophils” as they have the same origin, differentiation, cell surface markers, and phenotypes of classical neutrophils.^30^, ^152^ As described previously, MDSCs are described as an immature state of neutrophils or monocytes known for their immunosuppressive abilities and are often considered as a functional state of myeloid cells rather than a distinct cell type. However, cells in this immunosuppressive functional state are generalized as MDSCs. In mice, MDSCs have been shown to infiltrate several types of tumors and to support tumor invasion and metastasis^153^ via suppression of anti‐tumor immune responses.^154^ The immunosuppressive activities of the MDSC make them a potent hindrance to immunotherapeutic strategies and thus make them a highly appropriate target. Approaches for targeting MDSCs, including immature immunosuppressive neutrophils, include selective chemotherapeutic elimination of these cells,^155^ inhibition of MDSC‐mediated suppression,^156^ and inhibiting the accumulation of MDSC in the TME.^154^ As comprehensively reviewed by Wu et al., current clinical therapies targeting MDSCs are primarily centered on four key aspects^154^: (i) depletion of MDSCs including the use of chemotherapy such as gemcitabine and paclitaxel and antibody‐drug conjugates against CD33 (a common target for MDSCs),^134^ (ii) differentiation of MDSCs into mature myeloid and/or dendritic cells including the use of all‐trans retinoic acid^135^ and toll‐like receptor agonists,^157^ (iii) inhibition of the immunosuppressive activity of MDSCs by targeting critical biochemical pathways of MDSCs (such as by inhibiting ARG1, iNOS, COX2, PDE5, TGF‐β, and histone deacetylase inhibitors),^136^, ^137^, ^138^, ^139^, ^158^ and (iv) blockade of MDSCs expansion or activation by the blockade of chemokine receptors (CXCR2 and CXCR4) which have been shown to reduce MDSCs trafficking and tyrosine kinase inhibitors as well as STAT3 inhibitors which hold great promise as MDSC‐targeted immunotherapy.^140^, ^141^, ^142^, ^143^, ^144^, ^145^, ^159^

A caveat to this approach is that granulocytic MDSCs/immunosuppressive neutrophils and anti‐tumor/immunoactivating neutrophils share cell surface markers which requires targeting of the phenotype. If a tumor has both suppressive and anti‐tumor neutrophils within the same microenvironment, as previously described by our group,^66^ both populations would be depleted in targeting of cell surface markers. Thus, many studies are focusing on distinguishing overlapping populations for more targeted approaches.

### 8.4 CAR‐neutrophils

T cells and NK cells have been engineered to express CARs to boost their anti‐tumor effects for improved therapeutic potential.^160^, ^161^ Similarly, macrophages have been genetically equipped with CARs to improve their phagocytic ability.^7^, ^162^ These developments are revolutionizing the field of cancer immunotherapy and have stimulated interest in CAR engineering of neutrophils to enhance their anti‐tumor potency. In an early landmark study, Roberts et al.^146^ generated murine neutrophils with chimeric immune receptors and showed that neutrophils engineered to express zeta‐ and gamma‐chains of T‐cell receptors were able to perform target‐specific killing. However, neutrophil viability would need to be considered since they are terminally differentiated cells and have limited viability after isolation.^48^, ^163^, ^164^

To circumvent the short‐term viability of neutrophils, Chang et al., genetically engineered human pluripotent stem cells to generate glioblastoma‐specific CAR‐neutrophils which demonstrated potent and specific anti‐tumor effects both in vitro and in vivo in glioblastoma xenograft mouse model.^7^ In addition to significantly inhibiting tumor growth, these CAR‐neutrophils were reported to prolong the survival of the mice. Tumor cell killing by CAR‐neutrophils was mediated by phagocytosis, ROS production, and formation of NETs, thus exploiting the very arsenal by which neutrophils attack microbial pathogens. The fact that this strategy seems to drive anti‐tumor effects in the TME is particularly encouraging. These researchers recently used a CRISPR/Cas9‐mediated gene knock‐in strategy to generate CAR‐neutrophils which were then used to deliver glioblastoma microenvironment‐responsive nanodrugs. This approach termed as “combinatory chemo‐immunotherapy” by Chang et al. led to specific anti‐glioblastoma activities, specific delivery of nanodrugs to the tumor, and increased lifespan of tumor‐bearing mice.^147^

### 8.5 Neutrophil cooperation with T cells

A very recent study by Gungabeesoon et al. identified that in both mice and human lung cancers, neutrophil numbers significantly expand in tumors that have successful responses to immunotherapy.^165^ Upon administering forms of immunotherapy (e.g., anti‐CD40 treatment and anti‐PD‐1 treatment) they uncovered a state of neutrophils termed *Sell* ^*hi*^, referred to as ‘therapy‐elicited neutrophils’. These neutrophils appeared to be anti‐tumorigenic as blockade of this state of neutrophils in mice resulted in the loss of the therapeutic benefits. These therapy‐elicited neutrophils acquired an interferon gene signature in both mice and human patients, and this gene signature was deemed necessary for successful therapy. Furthermore, they characterized the neutrophil response to depend on dendritic cells, IL‐12 which activates cytotoxic T cells, and IFNγ (produced by said T cells). Remarkably, this therapy‐elicited systemic neutrophil response positively correlated with disease outcomes in lung cancer patients, hence emphasizing how the treatment‐induced reprogramming of neutrophils could enhance the anti‐tumor response.

Another recent seminal study conducted by Hirschhorn and the group has shed light on the critical role of neutrophils in T‐cell immunotherapies, particularly in their ability to eliminate tumor antigen escape variants.^166^ Over time, adoptive T‐cell transfer among other immunotherapies, can turn out to be unsuccessful as tumors evolve into antigen‐loss‐variant clones to evade the immune response. This group demonstrates that their melanoma‐specific CD4^+^ T‐cell therapy in combination with OX40 co‐stimulation of T cells or CTLA‐4 blockade makes it possible to overcome these escape variants and improve the effectiveness of the therapy.^166^, ^167^ Fascinatingly, during the early stages of this anti‐tumor immune response, CD4^+^ T cells play a crucial role in specifically recognizing the tumor. However, the complete eradication of the tumor was found to be dependent on neutrophils which express high levels of iNOS. Post‐immune checkpoint blockade treatment, they observed an extensive infiltration of activated neutrophils which were a distinct anti‐tumorigenic neutrophil subset, particularly in those with better response to the therapy. These neutrophils were regarded crucial for the efficacy of the treatment. This revolutionary discovery holds large implications for cancer immunotherapy, as it highlights the critical role of neutrophils in combination with T‐cell therapy. This paradigm shift underlines the importance of harnessing the collaborative potential of T cells and neutrophils to overcome immune evasion, thus forging new avenues for novel immunotherapeutic strategies that employ a variety of immune cells.

## 9 CONCLUSION

With an immense and ever‐growing body of data revealing the importance of neutrophils as drivers (or suppressors) of tumor progression and disease, there are three major considerations for translating the evidence emphasized here into clinically relevant therapies and improved patient outcomes. First, neutrophils are complicated and difficult to study because of the terminally differentiated status, reduced viability, sensitivity to premature activation, overlap with MDSCs, and species‐associated functional differences. Second, an abundance of early neutrophil cancer studies drove the field to believe that neutrophils are predominantly pro‐tumoral when, in fact, neutrophils are as diverse in function as their innate immune “partners‐in‐crime”, macrophages. Third, in many disease contexts, neutrophils exhibit anti‐tumor phenotypes and can cooperate with other immune cells, including adaptive cells, allowing them to be reprogrammed in a tissue setting, as needed.

In this review, we primarily focused on specific methods for enhancing neutrophil anti‐tumor immunity or targeting of neutrophil pro‐tumor responses. However, it is likely that these neutrophil‐targeted methods should be considered alongside current standard therapeutic approaches that cause the side effects of neutropenia, such as radiation and chemotherapy. Though neutropenia would be beneficial in scenarios in which pro‐tumor neutrophils are in abundance within the tumor, this effect would also deplete anti‐tumor neutrophils. To enhance the efficacy of neutrophil‐based therapy, it will be necessary to thoroughly consider the sequencing and scheduling in relation to other therapies, including anti‐cancer chemotherapy. In short, more investigation is needed; however, we are rapidly coming up with a renaissance of immunotherapy that may place neutrophils at the forefront of the next therapeutic breakthrough.

## AUTHOR CONTRIBUTIONS

**Sanjana Rajgopal:** Conceptualization (supporting); writing – original draft (equal); writing – review and editing (supporting). **Kosuke Nakano:** Conceptualization (supporting); writing – original draft (equal); writing – review and editing (supporting). **Leah M. Cook:** Conceptualization (lead); writing – original draft (supporting); writing – review and editing (lead).

## FUNDING INFORMATION

LMC was supported by a Research Scholar Grant (RSG‐19‐127‐01‐CSM) from the American Cancer Society, the Congressional Directed Medical Research Program, Prostate Cancer Research Program (W81XWH2110917), and the National Cancer Institute (1R01CA274605‐01).

## CONFLICT OF INTEREST STATEMENT

The authors have nothing to disclose.

## Data Availability

The data that support the findings of this study are available from the corresponding author upon reasonable request.

## References

[1] Zhang Y , Zhang Z . The history and advances in cancer immunotherapy: understanding the characteristics of tumor‐infiltrating immune cells and their therapeutic implications. Cell Mol Immunol. 2020;17:807‐821. doi:10.1038/s41423-020-0488-6 32612154 PMC7395159

[2] Labani‐Motlagh A , Ashja‐Mahdavi M , Loskog A . The tumor microenvironment: a milieu hindering and obstructing antitumor immune responses. Front Immunol. 2020;11:940. doi:10.3389/fimmu.2020.00940 32499786 PMC7243284

[3] Rohaan MW , Wilgenhof S , Haanen J . Adoptive cellular therapies: the current landscape. Virchows Arch. 2019;474:449‐461. doi:10.1007/s00428-018-2484-0 30470934 PMC6447513

[4] Mehrabadi AZ , Ranjbar R , Farzanehpour M , et al. Therapeutic potential of CAR T cell in malignancies: a scoping review. Biomed Pharmacother. 2022;146:112512. doi:10.1016/j.biopha.2021.112512 34894519

[5] Sloas C , Gill S , Klichinsky M . Engineered CAR‐macrophages as adoptive immunotherapies for solid tumors. Front Immunol. 2021;12:783305. doi:10.3389/fimmu.2021.783305 34899748 PMC8652144

[6] Li H , Song W , Li Z , Zhang M . Preclinical and clinical studies of CAR‐NK‐cell therapies for malignancies. Front Immunol. 2022;13:992232. doi:10.3389/fimmu.2022.992232 36353643 PMC9637940

[7] Chang Y , Syahirah R , Wang X , et al. Engineering chimeric antigen receptor neutrophils from human pluripotent stem cells for targeted cancer immunotherapy. Cell Rep. 2022;40:111128. doi:10.1016/j.celrep.2022.111128 35858579 PMC9327527

[8] Igarashi Y , Sasada T . Cancer vaccines: toward the next breakthrough in cancer immunotherapy. J Immunol Res. 2020;2020:5825401. doi:10.1155/2020/5825401 33282961 PMC7685825

[9] Anassi E , Ndefo UA . Sipuleucel‐T (provenge) injection: the first immunotherapy agent (vaccine) for hormone‐refractory prostate cancer. P T. 2011;36:197‐202. 21572775 PMC3086121

[10] Robert C . A decade of immune‐checkpoint inhibitors in cancer therapy. Nat Commun. 2020;11:3801. doi:10.1038/s41467-020-17670-y 32732879 PMC7393098

[11] Liu J , Chen Z , Li Y , Zhao W , Wu J , Zhang Z . PD‐1/PD‐L1 checkpoint inhibitors in tumor immunotherapy. Front Pharmacol. 2021;12:731798. doi:10.3389/fphar.2021.731798 34539412 PMC8440961

[12] Sharpe AH , Freeman GJ . The B7‐CD28 superfamily. Nat Rev Immunol. 2002;2:116‐126. doi:10.1038/nri727 11910893

[13] Kennedy A , Waters E , Rowshanravan B , et al. Differences in CD80 and CD86 transendocytosis reveal CD86 as a key target for CTLA‐4 immune regulation. Nat Immunol. 2022;23:1365‐1378. doi:10.1038/s41590-022-01289-w 35999394 PMC9477731

[14] Xin Yu J , Hubbard‐Lucey VM , Tang J . Immuno‐oncology drug development goes global. Nat Rev Drug Discov. 2019;18:899‐900. doi:10.1038/d41573-019-00167-9 31780841

[15] Liebl MC , Hofmann TG . Identification of responders to immune checkpoint therapy: which biomarkers have the highest value? J Eur Acad Dermatol Venereol. 2019;33(Suppl 8):52‐56. doi:10.1111/jdv.15992 31833606

[16] Paucek RD , Baltimore D , Li G . The cellular immunotherapy revolution: arming the immune system for precision therapy. Trends Immunol. 2019;40:292‐309. doi:10.1016/j.it.2019.02.002 30871979

[17] Galon J , Bruni D . Approaches to treat immune hot, altered and cold tumours with combination immunotherapies. Nat Rev Drug Discov. 2019;18:197‐218. doi:10.1038/s41573-018-0007-y 30610226

[18] Jardim DL , Goodman A , de Melo Gagliato D , Kurzrock R . The challenges of tumor mutational burden as an immunotherapy biomarker. Cancer Cell. 2021;39:154‐173. doi:10.1016/j.ccell.2020.10.001 33125859 PMC7878292

[19] Wolchok JD , Chiarion‐Sileni V , Gonzalez R , et al. Overall survival with combined nivolumab and ipilimumab in advanced melanoma. N Engl J Med. 2017;377:1345‐1356. doi:10.1056/NEJMoa1709684 28889792 PMC5706778

[20] Motzer RJ , Tannir NM , McDermott DF , et al. Nivolumab plus ipilimumab versus sunitinib in advanced renal‐cell carcinoma. N Engl J Med. 2018;378:1277‐1290. doi:10.1056/NEJMoa1712126 29562145 PMC5972549

[21] Shields MD , Marin‐Acevedo JA , Pellini B . Immunotherapy for advanced non‐small cell lung cancer: a decade of progress. Am Soc Clin Oncol Educ Book. 2021;41:1‐23. doi:10.1200/EDBK_321483 33979196

[22] de Bono JS , Guo C , Gurel B , et al. Prostate carcinogenesis: inflammatory storms. Nat Rev Cancer. 2020;20:455‐469. doi:10.1038/s41568-020-0267-9 32546840

[23] Krueger TE , Thorek DLJ , Meeker AK , Isaacs JT , Brennen WN . Tumor‐infiltrating mesenchymal stem cells: drivers of the immunosuppressive tumor microenvironment in prostate cancer? Prostate. 2019;79:320‐330. doi:10.1002/pros.23738 30488530 PMC6549513

[24] Mantovani A , Marchesi F , Malesci A , Laghi L , Allavena P . Tumour‐associated macrophages as treatment targets in oncology. Nat Rev Clin Oncol. 2017;14:399‐416. doi:10.1038/nrclinonc.2016.217 28117416 PMC5480600

[25] Tucker SL , Sarr D , Rada B . Granulocytic myeloid‐derived suppressor cells in cystic fibrosis. Front Immunol. 2021;12:745326. doi:10.3389/fimmu.2021.745326 34621276 PMC8490623

[26] Villanueva E , Yalavarthi S , Berthier CC , et al. Netting neutrophils induce endothelial damage, infiltrate tissues, and expose immunostimulatory molecules in systemic lupus erythematosus. J Immunol. 2011;187:538‐552. doi:10.4049/jimmunol.1100450 21613614 PMC3119769

[27] Redd PS , Ibrahim ML , Klement JD , et al. SETD1B activates iNOS expression in myeloid‐derived suppressor cells. Cancer Res. 2017;77:2834‐2843. doi:10.1158/0008-5472.CAN-16-2238 28381543 PMC5495112

[28] Otani Y , Yoo JY , Lewis CT , et al. NOTCH‐induced MDSC recruitment after oHSV virotherapy in CNS cancer models modulates antitumor immunotherapy. Clin Cancer Res. 2022;28:1460‐1473. doi:10.1158/1078-0432.CCR-21-2347 35022322 PMC8976724

[29] Grover A , Sanseviero E , Timosenko E , Gabrilovich DI . Myeloid‐derived suppressor cells: a propitious road to clinic. Cancer Discov. 2021;11:2693‐2706. doi:10.1158/2159-8290.CD-21-0764 34635571

[30] Bronte V , Brandau S , Chen SH , et al. Recommendations for myeloid‐derived suppressor cell nomenclature and characterization standards. Nat Commun. 2016;7:12150. doi:10.1038/ncomms12150 27381735 PMC4935811

[31] Hedrick CC , Malanchi I . Neutrophils in cancer: heterogeneous and multifaceted. Nat Rev Immunol. 2022;22:173‐187. doi:10.1038/s41577-021-00571-6 34230649

[32] Coffelt SB , Wellenstein MD , de Visser KE . Neutrophils in cancer: neutral no more. Nat Rev Cancer. 2016;16:431‐446. doi:10.1038/nrc.2016.52 27282249

[33] Casbon AJ , Reynaud D , Park C , et al. Invasive breast cancer reprograms early myeloid differentiation in the bone marrow to generate immunosuppressive neutrophils. Proc Natl Acad Sci U S A. 2015;112:E566‐E575. doi:10.1073/pnas.1424927112 25624500 PMC4330753

[34] Park J , Wysocki RW , Amoozgar Z , et al. Cancer cells induce metastasis‐supporting neutrophil extracellular DNA traps. Sci Transl Med. 2016;8:361ra138. doi:10.1126/scitranslmed.aag1711 PMC5550900 27798263

[35] Wang Y , Xu M , Sun J , et al. Glycolytic neutrophils accrued in the spleen compromise anti‐tumour T cell immunity in breast cancer. Nat Metab. 2023;5:1408‐1422. doi:10.1038/s42255-023-00853-4 37563468

[36] Akinci Ozyurek B , Sahin Ozdemirel T , Buyukyaylaci Ozden S , Erdogan Y , Kaplan B , Kaplan T . Prognostic value of the neutrophil to lymphocyte ratio (NLR) in lung cancer cases. Asian Pac J Cancer Prev. 2017;18:1417‐1421. doi:10.22034/APJCP.2017.18.5.1417 28612596 PMC5555556

[37] Zhang W , Shen Y , Huang H , et al. A Rosetta Stone for breast cancer: prognostic value and dynamic regulation of neutrophil in tumor microenvironment. Front Immunol. 2020;11:1779. doi:10.3389/fimmu.2020.01779 32849640 PMC7426521

[38] Templeton AJ , McNamara MG , Seruga B , et al. Prognostic role of neutrophil‐to‐lymphocyte ratio in solid tumors: a systematic review and meta‐analysis. J Natl Cancer Inst. 2014;106:dju124. doi:10.1093/jnci/dju124 24875653

[39] Rosales C , Lowell CA , Schnoor M , Uribe‐Querol E . Neutrophils: their role in innate and adaptive immunity. J Immunol Res. 2017;2017:9748345. doi:10.1155/2017/9748345 29238732 PMC5697120

[40] Malech HL , Deleo FR , Quinn MT . The role of neutrophils in the immune system: an overview. Methods Mol Biol. 2014;1124:3‐10. doi:10.1007/978-1-62703-845-4_1 24504942 PMC6777345

[41] Byun JS , Gardner K . Wounds that will not heal: pervasive cellular reprogramming in cancer. Am J Pathol. 2013;182:1055‐1064. doi:10.1016/j.ajpath.2013.01.009 23438473 PMC3657619

[42] Dvorak HF . Tumors: wounds that do not heal‐redux. Cancer Immunol Res. 2015;3:1‐11. doi:10.1158/2326-6066.CIR-14-0209 25568067 PMC4288010

[43] Marone G , Gambardella AR , Mattei F , Mancini J , Schiavoni G , Varricchi G . Basophils in tumor microenvironment and surroundings. Adv Exp Med Biol. 2020;1224:21‐34. doi:10.1007/978-3-030-35723-8_2 32036602

[44] Marone G , Schroeder JT , Mattei F , et al. Is there a role for basophils in cancer? Front Immunol. 2020;11:2103. doi:10.3389/fimmu.2020.02103 33013885 PMC7505934

[45] Blomberg OS , Spagnuolo L , Garner H , et al. IL‐5‐producing CD4(+) T cells and eosinophils cooperate to enhance response to immune checkpoint blockade in breast cancer. Cancer Cell. 2023;41:106‐123.e10. doi:10.1016/j.ccell.2022.11.014 36525971

[46] Varricchi G , Galdiero MR , Loffredo S , et al. Eosinophils: the unsung heroes in cancer? Onco Targets Ther. 2018;7:e1393134. doi:10.1080/2162402X.2017.1393134 PMC5749653 29308325

[47] Gierlikowska B , Stachura A , Gierlikowski W , Demkow U . Phagocytosis, degranulation and extracellular traps release by neutrophils‐the current knowledge, pharmacological modulation and future prospects. Front Pharmacol. 2021;12:666732. doi:10.3389/fphar.2021.666732 34017259 PMC8129565

[48] Kolaczkowska E , Kubes P . Neutrophil recruitment and function in health and inflammation. Nat Rev Immunol. 2013;13:159‐175. doi:10.1038/nri3399 23435331

[49] Uribe‐Querol E , Rosales C . Neutrophils in cancer: two sides of the same coin. J Immunol Res. 2015;2015:983698. doi:10.1155/2015/983698 26819959 PMC4706937

[50] Lin Y , Cheng L , Liu Y , et al. Intestinal epithelium‐derived BATF3 promotes colitis‐associated colon cancer through facilitating CXCL5‐mediated neutrophils recruitment. Mucosal Immunol. 2021;14:187‐198. doi:10.1038/s41385-020-0297-3 32467604

[51] Yu PF , Huang Y , Han YY , et al. TNFalpha‐activated mesenchymal stromal cells promote breast cancer metastasis by recruiting CXCR2(+) neutrophils. Oncogene. 2017;36:482‐490. doi:10.1038/onc.2016.217 27375023 PMC5290040

[52] Swierczak A , Mouchemore KA , Hamilton JA , Anderson RL . Neutrophils: important contributors to tumor progression and metastasis. Cancer Metastasis Rev. 2015;34:735‐751. doi:10.1007/s10555-015-9594-9 26361774

[53] SenGupta S , Hein LE , Parent CA . The recruitment of neutrophils to the tumor microenvironment is regulated by multiple mediators. Front Immunol. 2021;12:734188. doi:10.3389/fimmu.2021.734188 34567000 PMC8461236

[54] Teijeira A , Garasa S , Ochoa MC , et al. IL8, neutrophils, and NETs in a collusion against cancer immunity and immunotherapy. Clin Cancer Res. 2021;27:2383‐2393. doi:10.1158/1078-0432.CCR-20-1319 33376096

[55] David JM , Dominguez C , Hamilton DH , Palena C . The IL‐8/IL‐8R axis: a double agent in tumor immune resistance. Vaccines (Basel). 2016;4:22. doi:10.3390/vaccines4030022 27348007 PMC5041016

[56] Xiao Y , Cong M , Li J , et al. Cathepsin C promotes breast cancer lung metastasis by modulating neutrophil infiltration and neutrophil extracellular trap formation. Cancer Cell. 2021;39:423‐437.e7. doi:10.1016/j.ccell.2020.12.012 33450198

[57] Fridlender ZG , Sun J , Kim S , et al. Polarization of tumor‐associated neutrophil phenotype by TGF‐beta: “N1” versus “N2” TAN. Cancer Cell. 2009;16:183‐194. doi:10.1016/j.ccr.2009.06.017 19732719 PMC2754404

[58] SenGupta S , Hein LE , Xu Y , et al. Triple‐negative breast cancer cells recruit neutrophils by secreting TGF‐beta and CXCR2 ligands. Front Immunol. 2021;12:659996. doi:10.3389/fimmu.2021.659996 33912188 PMC8071875

[59] Wu L , Awaji M , Saxena S , Varney ML , Sharma B , Singh RK . IL‐17‐CXC chemokine receptor 2 axis facilitates breast cancer progression by up‐regulating neutrophil recruitment. Am J Pathol. 2020;190:222‐233. doi:10.1016/j.ajpath.2019.09.016 31654638 PMC6943375

[60] Muller A , Homey B , Soto H , et al. Involvement of chemokine receptors in breast cancer metastasis. Nature. 2001;410:50‐56. doi:10.1038/35065016 11242036

[61] Chen E , Yu J . The role and metabolic adaptations of neutrophils in premetastatic niches. Biomark Res. 2023;11:50. doi:10.1186/s40364-023-00493-6 37158964 PMC10169509

[62] Seubert B , Grunwald B , Kobuch J , et al. Tissue inhibitor of metalloproteinases (TIMP)‐1 creates a premetastatic niche in the liver through SDF‐1/CXCR4‐dependent neutrophil recruitment in mice. Hepatology. 2015;61:238‐248. doi:10.1002/hep.27378 25131778 PMC4280301

[63] Finisguerra V , Di Conza G , Di Matteo M , et al. MET is required for the recruitment of anti‐tumoural neutrophils. Nature. 2015;522:349‐353. doi:10.1038/nature14407 25985180 PMC4594765

[64] Granot Z , Henke E , Comen EA , King TA , Norton L , Benezra R . Tumor entrained neutrophils inhibit seeding in the premetastatic lung. Cancer Cell. 2011;20:300‐314. doi:10.1016/j.ccr.2011.08.012 21907922 PMC3172582

[65] Yan J , Kloecker G , Fleming C , et al. Human polymorphonuclear neutrophils specifically recognize and kill cancerous cells. Onco Targets Ther. 2014;3:e950163. doi:10.4161/15384101.2014.950163 PMC4292216 25610737

[66] Costanzo‐Garvey DL , Keeley T , Case AJ , et al. Neutrophils are mediators of metastatic prostate cancer progression in bone. Cancer Immunol Immunother. 2020;69:1113‐1130. doi:10.1007/s00262-020-02527-6 32114681 PMC7230043

[67] Costanzo‐Garvey DL , Case AJ , Watson GF , et al. Prostate cancer addiction to oxidative stress defines sensitivity to anti‐tumor neutrophils. Clin Exp Metastasis. 2022;39:641‐659. doi:10.1007/s10585-022-10170-x 35604506 PMC9338904

[68] Gershkovitz M , Caspi Y , Fainsod‐Levi T , et al. TRPM2 mediates neutrophil killing of disseminated tumor cells. Cancer Res. 2018;78:2680‐2690. doi:10.1158/0008-5472.CAN-17-3614 29490946

[69] Zhong J , Li Q , Luo H , Holmdahl R . Neutrophil‐derived reactive oxygen species promote tumor colonization. Commun Biol. 2021;4:865. doi:10.1038/s42003-021-02376-8 34257370 PMC8277858

[70] Rice CM , Davies LC , Subleski JJ , et al. Tumour‐elicited neutrophils engage mitochondrial metabolism to circumvent nutrient limitations and maintain immune suppression. Nat Commun. 2018;9:5099. doi:10.1038/s41467-018-07505-2 30504842 PMC6269473

[71] Brinkmann V , Reichard U , Goosmann C , et al. Neutrophil extracellular traps kill bacteria. Science. 2004;303:1532‐1535. doi:10.1126/science.1092385 15001782

[72] Neuenfeldt F , Schumacher JC , Grieshaber‐Bouyer R , et al. Inflammation induces pro‐NETotic neutrophils via TNFR2 signaling. Cell Rep. 2022;39:110710. doi:10.1016/j.celrep.2022.110710 35443164

[73] Azzouz D , Khan MA , Palaniyar N . ROS induces NETosis by oxidizing DNA and initiating DNA repair. Cell Death Discov. 2021;7:113. doi:10.1038/s41420-021-00491-3 34001856 PMC8128883

[74] Vorobjeva NV , Chernyak BV . NETosis: molecular mechanisms, role in physiology and pathology. Biochemistry (Mosc). 2020;85:1178‐1190. doi:10.1134/S0006297920100065 33202203 PMC7590568

[75] Yang LY , Luo Q , Lu L , et al. Increased neutrophil extracellular traps promote metastasis potential of hepatocellular carcinoma via provoking tumorous inflammatory response. J Hematol Oncol. 2020;13:3. doi:10.1186/s13045-019-0836-0 31907001 PMC6945602

[76] Jung HS , Gu J , Kim JE , Nam Y , Song JW , Kim HK . Cancer cell‐induced neutrophil extracellular traps promote both hypercoagulability and cancer progression. PLoS One. 2019;14:e0216055. doi:10.1371/journal.pone.0216055 31034495 PMC6488070

[77] Inoue M , Nakashima R , Enomoto M , et al. Plasma redox imbalance caused by albumin oxidation promotes lung‐predominant NETosis and pulmonary cancer metastasis. Nat Commun. 2018;9:5116. doi:10.1038/s41467-018-07550-x 30504805 PMC6269536

[78] Cools‐Lartigue J , Spicer J , McDonald B , et al. Neutrophil extracellular traps sequester circulating tumor cells and promote metastasis. J Clin Invest. 2013;123:3446‐3458. doi:10.1172/JCI67484 23863628 PMC3726160

[79] Lee W , Ko SY , Mohamed MS , Kenny HA , Lengyel E , Naora H . Neutrophils facilitate ovarian cancer premetastatic niche formation in the omentum. J Exp Med. 2019;216:176‐194. doi:10.1084/jem.20181170 30567719 PMC6314534

[80] Tohme S , Yazdani HO , Al‐Khafaji AB , et al. Neutrophil extracellular traps promote the development and progression of liver metastases after surgical stress. Cancer Res. 2016;76:1367‐1380. doi:10.1158/0008-5472.CAN-15-1591 26759232 PMC4794393

[81] Yang L , Liu Q , Zhang X , et al. DNA of neutrophil extracellular traps promotes cancer metastasis via CCDC25. Nature. 2020;583:133‐138. doi:10.1038/s41586-020-2394-6 32528174

[82] Albrengues J , Shields MA , Ng D , et al. Neutrophil extracellular traps produced during inflammation awaken dormant cancer cells in mice. Science. 2018;361:aao4227. doi:10.1126/science.aao4227 PMC6777850 30262472

[83] Shahzad MH , Feng L , Su X , et al. Neutrophil extracellular traps in cancer therapy resistance. Cancers (Basel). 2022;14:1359. doi:10.3390/cancers14051359 35267667 PMC8909607

[84] Ramachandran IR , Condamine T , Lin C , et al. Bone marrow PMN‐MDSCs and neutrophils are functionally similar in protection of multiple myeloma from chemotherapy. Cancer Lett. 2016;371:117‐124. doi:10.1016/j.canlet.2015.10.040 26639197 PMC4919899

[85] Mousset A , Lecorgne E , Bourget I , et al. Neutrophil extracellular traps formed during chemotherapy confer treatment resistance via TGF‐beta activation. Cancer Cell. 2023;41:757‐775.e10. doi:10.1016/j.ccell.2023.03.008 37037615 PMC10228050

[86] Shinde‐Jadhav S , Mansure JJ , Rayes RF , et al. Role of neutrophil extracellular traps in radiation resistance of invasive bladder cancer. Nat Commun. 2021;12:2776. doi:10.1038/s41467-021-23086-z 33986291 PMC8119713

[87] Teijeira A , Garasa S , Gato M , et al. CXCR1 and CXCR2 chemokine receptor agonists produced by tumors induce neutrophil extracellular traps that interfere with immune cytotoxicity. Immunity. 2020;52:856‐871.e8. doi:10.1016/j.immuni.2020.03.001 32289253

[88] Lacy P . Mechanisms of degranulation in neutrophils. Allergy Asthma Clin Immunol. 2006;2:98‐108. doi:10.1186/1710-1492-2-3-98 20525154 PMC2876182

[89] Cassatella MA , Ostberg NK , Tamassia N , Soehnlein O . Biological roles of neutrophil‐derived granule proteins and cytokines. Trends Immunol. 2019;40:648‐664. doi:10.1016/j.it.2019.05.003 31155315

[90] Borregaard N , Lollike K , Kjeldsen L , et al. Human neutrophil granules and secretory vesicles. Eur J Haematol. 1993;51:187‐198. doi:10.1111/j.1600-0609.1993.tb00629.x 8243606

[91] Ardi VC , Kupriyanova TA , Deryugina EI , Quigley JP . Human neutrophils uniquely release TIMP‐free MMP‐9 to provide a potent catalytic stimulator of angiogenesis. Proc Natl Acad Sci U S A. 2007;104:20262‐20267. doi:10.1073/pnas.0706438104 18077379 PMC2154419

[92] Deryugina EI , Zajac E , Juncker‐Jensen A , Kupriyanova TA , Welter L , Quigley JP . Tissue‐infiltrating neutrophils constitute the major in vivo source of angiogenesis‐inducing MMP‐9 in the tumor microenvironment. Neoplasia. 2014;16:771‐788. doi:10.1016/j.neo.2014.08.013 25379015 PMC4212255

[93] Bekes EM , Schweighofer B , Kupriyanova TA , et al. Tumor‐recruited neutrophils and neutrophil TIMP‐free MMP‐9 regulate coordinately the levels of tumor angiogenesis and efficiency of malignant cell intravasation. Am J Pathol. 2011;179:1455‐1470. doi:10.1016/j.ajpath.2011.05.031 21741942 PMC3157227

[94] Sato T , Takahashi S , Mizumoto T , et al. Neutrophil elastase and cancer. Surg Oncol. 2006;15:217‐222. doi:10.1016/j.suronc.2007.01.003 17320378

[95] Akizuki M , Fukutomi T , Takasugi M , et al. Prognostic significance of immunoreactive neutrophil elastase in human breast cancer: long‐term follow‐up results in 313 patients. Neoplasia. 2007;9:260‐264. doi:10.1593/neo.06808 17401466 PMC1838583

[96] Inada M , Yamashita J , Ogawa M . Neutrophil elastase inhibitor (ONO‐5046‐Na) inhibits the growth of human lung cancer cell lines transplanted into severe combined immunodeficiency (SCID) mice. Res Commun Mol Pathol Pharmacol. 1997;97:229‐232. 9344234

[97] Gong L , Cumpian AM , Caetano MS , et al. Promoting effect of neutrophils on lung tumorigenesis is mediated by CXCR2 and neutrophil elastase. Mol Cancer. 2013;12:154. doi:10.1186/1476-4598-12-154 24321240 PMC3923587

[98] Lerman I , Hammes SR . Neutrophil elastase in the tumor microenvironment. Steroids. 2018;133:96‐101. doi:10.1016/j.steroids.2017.11.006 29155217 PMC5870895

[99] Ho AS , Chen CH , Cheng CC , et al. Neutrophil elastase as a diagnostic marker and therapeutic target in colorectal cancers. Oncotarget. 2014;5:473‐480. doi:10.18632/oncotarget.1631 24457622 PMC3964222

[100] Houghton AM , Rzymkiewicz DM , Ji H , et al. Neutrophil elastase‐mediated degradation of IRS‐1 accelerates lung tumor growth. Nat Med. 2010;16:219‐223. doi:10.1038/nm.2084 20081861 PMC2821801

[101] El Rayes T , Catena R , Lee S , et al. Lung inflammation promotes metastasis through neutrophil protease‐mediated degradation of Tsp‐1. Proc Natl Acad Sci U S A. 2015;112:16000‐16005. doi:10.1073/pnas.1507294112 26668367 PMC4703007

[102] Cui C , Chakraborty K , Tang XA , et al. Neutrophil elastase selectively kills cancer cells and attenuates tumorigenesis. Cell. 2021;184:3163‐3177.e21. doi:10.1016/j.cell.2021.04.016 33964209 PMC10712736

[103] Peng B , Hu J , Fu X . ELANE: an emerging lane to selective anticancer therapy. Signal Transduct Target Ther. 2021;6:358. doi:10.1038/s41392-021-00766-2 34599140 PMC8486838

[104] Zeng MY , Miralda I , Armstrong CL , Uriarte SM , Bagaitkar J . The roles of NADPH oxidase in modulating neutrophil effector responses. Mol Oral Microbiol. 2019;34:27‐38. doi:10.1111/omi.12252 30632295 PMC6935359

[105] Clancy DM , Henry CM , Sullivan GP , Martin SJ . Neutrophil extracellular traps can serve as platforms for processing and activation of IL‐1 family cytokines. FEBS J. 2017;284:1712‐1725. doi:10.1111/febs.14075 28374518

[106] Pyrillou K , Burzynski LC , Clarke MCH . Alternative pathways of IL‐1 activation, and its role in health and disease. Front Immunol. 2020;11:613170. doi:10.3389/fimmu.2020.613170 33391283 PMC7775495

[107] Valadez‐Cosmes P , Raftopoulou S , Mihalic ZN , Marsche G , Kargl J . Myeloperoxidase: growing importance in cancer pathogenesis and potential drug target. Pharmacol Ther. 2022;236:108052. doi:10.1016/j.pharmthera.2021.108052 34890688

[108] Panagopoulos V , Leach DA , Zinonos I , et al. Inflammatory peroxidases promote breast cancer progression in mice via regulation of the tumour microenvironment. Int J Oncol. 2017;50:1191‐1200. doi:10.3892/ijo.2017.3883 28260049

[109] Liu TW , Gammon ST , Yang P , Ma W , Wang J , Piwnica‐Worms D . Inhibition of myeloperoxidase enhances immune checkpoint therapy for melanoma. J Immunother Cancer. 2023;11:e005837. doi:10.1136/jitc-2022-005837 36805920 PMC9944647

[110] Rosales C . Neutrophil: a cell with many roles in inflammation or several cell types? Front Physiol. 2018;9:113. doi:10.3389/fphys.2018.00113 29515456 PMC5826082

[111] Jaumouille V , Waterman CM . Physical constraints and forces involved in phagocytosis. Front Immunol. 2020;11:1097. doi:10.3389/fimmu.2020.01097 32595635 PMC7304309

[112] Ustyanovska Avtenyuk N , Visser N , Bremer E , Wiersma VR . The neutrophil: the underdog that packs a punch in the fight against cancer. Int J Mol Sci. 2020;21:17820. doi:10.3390/ijms21217820 PMC7659937 33105656

[113] Matlung HL , Babes L , Zhao XW , et al. Neutrophils kill antibody‐opsonized cancer cells by trogoptosis. Cell Rep. 2018;23:3946‐3959.e6. doi:10.1016/j.celrep.2018.05.082 29949776

[114] Stockmeyer B , Beyer T , Neuhuber W , et al. Polymorphonuclear granulocytes induce antibody‐dependent apoptosis in human breast cancer cells. J Immunol. 2003;171:5124‐5129. doi:10.4049/jimmunol.171.10.5124 14607911

[115] Xiong S , Dong L , Cheng L . Neutrophils in cancer carcinogenesis and metastasis. J Hematol Oncol. 2021;14:173. doi:10.1186/s13045-021-01187-y 34674757 PMC8529570

[116] Hampton HR , Bailey J , Tomura M , Brink R , Chtanova T . Microbe‐dependent lymphatic migration of neutrophils modulates lymphocyte proliferation in lymph nodes. Nat Commun. 2015;6:7139. doi:10.1038/ncomms8139 25972253 PMC4479041

[117] Mysore V , Cullere X , Mears J , et al. FcgammaR engagement reprograms neutrophils into antigen cross‐presenting cells that elicit acquired anti‐tumor immunity. Nat Commun. 2021;12:4791. doi:10.1038/s41467-021-24591-x 34373452 PMC8352912

[118] Puga I , Cols M , Barra CM , et al. B cell‐helper neutrophils stimulate the diversification and production of immunoglobulin in the marginal zone of the spleen. Nat Immunol. 2011;13:170‐180. doi:10.1038/ni.2194 22197976 PMC3262910

[119] Singhal S , Bhojnagarwala PS , O'Brien S , et al. Origin and role of a subset of tumor‐associated neutrophils with antigen‐presenting cell features in early‐stage human lung cancer. Cancer Cell. 2016;30:120‐135. doi:10.1016/j.ccell.2016.06.001 27374224 PMC4945447

[120] Pylaeva E , Korschunow G , Spyra I , et al. During early stages of cancer, neutrophils initiate anti‐tumor immune responses in tumor‐draining lymph nodes. Cell Rep. 2022;40:111171. doi:10.1016/j.celrep.2022.111171 35977505

[121] Pylaeva E , Ozel I , Squire A , et al. B‐helper neutrophils in regional lymph nodes correlate with improved prognosis in patients with head and neck cancer. Cancers (Basel). 2021;13:23092. doi:10.3390/cancers13123092 PMC8234083 34205654

[122] Xiao Y , Yu D . Tumor microenvironment as a therapeutic target in cancer. Pharmacol Ther. 2021;221:107753. doi:10.1016/j.pharmthera.2020.107753 33259885 PMC8084948

[123] van den Berg JH , Heemskerk B , van Rooij N , et al. Tumor infiltrating lymphocytes (TIL) therapy in metastatic melanoma: boosting of neoantigen‐specific T cell reactivity and long‐term follow‐up. J Immunother Cancer. 2020;8:e000848. doi:10.1136/jitc-2020-000848 32753545 PMC7406109

[124] Chen Y , McAndrews KM , Kalluri R . Clinical and therapeutic relevance of cancer‐associated fibroblasts. Nat Rev Clin Oncol. 2021;18:792‐804. doi:10.1038/s41571-021-00546-5 34489603 PMC8791784

[125] Pan Y , Yu Y , Wang X , Zhang T . Tumor‐associated macrophages in tumor immunity. Front Immunol. 2020;11:583084. doi:10.3389/fimmu.2020.583084 33365025 PMC7751482

[126] Fix SM , Forget MA , Sakellariou‐Thompson D , et al. CRISPR‐mediated TGFBR2 knockout renders human ovarian cancer tumor‐infiltrating lymphocytes resistant to TGF‐beta signaling. J Immunother Cancer. 2022;10:e003750. doi:10.1136/jitc-2021-003750 35882447 PMC9330322

[127] Tanaka A , Sakaguchi S . Regulatory T cells in cancer immunotherapy. Cell Res. 2017;27:109‐118. doi:10.1038/cr.2016.151 27995907 PMC5223231

[128] Mizuno R , Kawada K , Itatani Y , Ogawa R , Kiyasu Y , Sakai Y . The role of tumor‐associated neutrophils in colorectal cancer. Int J Mol Sci. 2019;20:529. doi:10.3390/ijms20030529 30691207 PMC6386937

[129] Qin F , Liu X , Chen J , et al. Anti‐TGF‐beta attenuates tumor growth via polarization of tumor associated neutrophils towards an anti‐tumor phenotype in colorectal cancer. J Cancer. 2020;11:2580‐2592. doi:10.7150/jca.38179 32201528 PMC7066015

[130] Szczerba BM , Castro‐Giner F , Vetter M , et al. Neutrophils escort circulating tumour cells to enable cell cycle progression. Nature. 2019;566:553‐557. doi:10.1038/s41586-019-0915-y 30728496

[131] Linde IL , Prestwood TR , Qiu J , et al. Neutrophil‐activating therapy for the treatment of cancer. Cancer Cell. 2023;41:356‐372.e10. doi:10.1016/j.ccell.2023.01.002 36706760 PMC9968410

[132] Steele CW , Karim SA , Leach JDG , et al. CXCR2 inhibition profoundly suppresses metastases and augments immunotherapy in pancreatic ductal adenocarcinoma. Cancer Cell. 2016;29:832‐845. doi:10.1016/j.ccell.2016.04.014 27265504 PMC4912354

[133] Jamieson T , Clarke M , Steele CW , et al. Inhibition of CXCR2 profoundly suppresses inflammation‐driven and spontaneous tumorigenesis. J Clin Invest. 2012;122:3127‐3144. doi:10.1172/JCI61067 22922255 PMC3428079

[134] Fultang L , Panetti S , Ng M , et al. MDSC targeting with gemtuzumab ozogamicin restores T cell immunity and immunotherapy against cancers. EBioMedicine. 2019;47:235‐246. doi:10.1016/j.ebiom.2019.08.025 31462392 PMC6796554

[135] Nefedova Y , Fishman M , Sherman S , Wang X , Beg AA , Gabrilovich DI . Mechanism of all‐trans retinoic acid effect on tumor‐associated myeloid‐derived suppressor cells. Cancer Res. 2007;67:11021‐11028. doi:10.1158/0008-5472.CAN-07-2593 18006848

[136] Yu SJ , Ma C , Heinrich B , et al. Targeting the crosstalk between cytokine‐induced killer cells and myeloid‐derived suppressor cells in hepatocellular carcinoma. J Hepatol. 2019;70:449‐457. doi:10.1016/j.jhep.2018.10.040 30414862 PMC6380944

[137] Tomita Y , Lee MJ , Lee S , et al. The interplay of epigenetic therapy and immunity in locally recurrent or metastatic estrogen receptor‐positive breast cancer: correlative analysis of ENCORE 301, a randomized, placebo‐controlled phase II trial of exemestane with or without entinostat. Onco Targets Ther. 2016;5:e1219008. doi:10.1080/2162402X.2016.1219008 PMC5139687 27999738

[138] Fujita M , Kohanbash G , Fellows‐Mayle W , et al. COX‐2 blockade suppresses gliomagenesis by inhibiting myeloid‐derived suppressor cells. Cancer Res. 2011;71:2664‐2674. doi:10.1158/0008-5472.CAN-10-3055 21324923 PMC3075086

[139] Steggerda SM , Bennett MK , Chen J , et al. Inhibition of arginase by CB‐1158 blocks myeloid cell‐mediated immune suppression in the tumor microenvironment. J Immunother Cancer. 2017;5:101. doi:10.1186/s40425-017-0308-4 29254508 PMC5735564

[140] Hong D , Kurzrock R , Kim Y , et al. AZD9150, a next‐generation antisense oligonucleotide inhibitor of STAT3 with early evidence of clinical activity in lymphoma and lung cancer. Sci Transl Med. 2015;7:314ra185. doi:10.1126/scitranslmed.aac5272 PMC5279222 26582900

[141] Shastri A , Choudhary G , Teixeira M , et al. Antisense STAT3 inhibitor decreases viability of myelodysplastic and leukemic stem cells. J Clin Invest. 2018;128:5479‐5488. doi:10.1172/JCI120156 30252677 PMC6264739

[142] Ko JS , Zea AH , Rini BI , et al. Sunitinib mediates reversal of myeloid‐derived suppressor cell accumulation in renal cell carcinoma patients. Clin Cancer Res. 2009;15:2148‐2157. doi:10.1158/1078-0432.CCR-08-1332 19276286

[143] Highfill SL , Cui Y , Giles AJ , et al. Disruption of CXCR2‐mediated MDSC tumor trafficking enhances anti‐PD1 efficacy. Sci Transl Med. 2014;6:237ra267. doi:10.1126/scitranslmed.3007974 PMC6980372 24848257

[144] D'Alterio C , Buoncervello M , Ierano C , et al. Targeting CXCR4 potentiates anti‐PD‐1 efficacy modifying the tumor microenvironment and inhibiting neoplastic PD‐1. J Exp Clin Cancer Res. 2019;38:432. doi:10.1186/s13046-019-1420-8 31661001 PMC6819555

[145] Bockorny B , Semenisty V , Macarulla T , et al. BL‐8040, a CXCR4 antagonist, in combination with pembrolizumab and chemotherapy for pancreatic cancer: the COMBAT trial. Nat Med. 2020;26:878‐885. doi:10.1038/s41591-020-0880-x 32451495

[146] Roberts MR , Cooke KS , Tran AC , et al. Antigen‐specific cytolysis by neutrophils and NK cells expressing chimeric immune receptors bearing zeta or gamma signaling domains. J Immunol. 1998;161:375‐384. 9647246

[147] Chang Y , Cai X , Syahirah R , et al. CAR‐neutrophil mediated delivery of tumor‐microenvironment responsive nanodrugs for glioblastoma chemo‐immunotherapy. Nat Commun. 2023;14:2266. doi:10.1038/s41467-023-37872-4 37080958 PMC10119091

[148] Korbecki J , Kupnicka P , Chlubek M , Goracy J , Gutowska I , Baranowska‐Bosiacka I . CXCR2 receptor: regulation of expression, signal transduction, and involvement in cancer. Int J Mol Sci. 2022;23:168. doi:10.3390/ijms23042168 PMC8878198 35216283

[149] Blaisdell A , Crequer A , Columbus D , et al. Neutrophils oppose uterine epithelial carcinogenesis via debridement of hypoxic tumor cells. Cancer Cell. 2015;28:785‐799. doi:10.1016/j.ccell.2015.11.005 26678340 PMC4698345

[150] Landskron G , De la Fuente M , Thuwajit P , Thuwajit C , Hermoso MA . Chronic inflammation and cytokines in the tumor microenvironment. J Immunol Res. 2014;2014:149185. doi:10.1155/2014/149185 24901008 PMC4036716

[151] Souto JC , Vila L , Bru A . Polymorphonuclear neutrophils and cancer: intense and sustained neutrophilia as a treatment against solid tumors. Med Res Rev. 2011;31:311‐363. doi:10.1002/med.20185 19967776

[152] Zhou J , Nefedova Y , Lei A , Gabrilovich D . Neutrophils and PMN‐MDSC: their biological role and interaction with stromal cells. Semin Immunol. 2018;35:19‐28. doi:10.1016/j.smim.2017.12.004 29254756 PMC5866202

[153] De Cicco P , Ercolano G , Ianaro A . The new era of cancer immunotherapy: targeting myeloid‐derived suppressor cells to overcome immune evasion. Front Immunol. 2020;11:1680. doi:10.3389/fimmu.2020.01680 32849585 PMC7406792

[154] Wu Y , Yi M , Niu M , Mei Q , Wu K . Myeloid‐derived suppressor cells: an emerging target for anticancer immunotherapy. Mol Cancer. 2022;21:184. doi:10.1186/s12943-022-01657-y 36163047 PMC9513992

[155] Rivera Vargas T , Apetoh L . Can immunogenic chemotherapies relieve cancer cell resistance to immune checkpoint inhibitors? Front Immunol. 2019;10:1181. doi:10.3389/fimmu.2019.01181 31191545 PMC6548803

[156] Holmgaard RB , Zamarin D , Li Y , et al. Tumor‐expressed IDO recruits and activates MDSCs in a Treg‐dependent manner. Cell Rep. 2015;13:412‐424. doi:10.1016/j.celrep.2015.08.077 26411680 PMC5013825

[157] Shirota Y , Shirota H , Klinman DM . Intratumoral injection of CpG oligonucleotides induces the differentiation and reduces the immunosuppressive activity of myeloid‐derived suppressor cells. J Immunol. 2012;188:1592‐1599. doi:10.4049/jimmunol.1101304 22231700 PMC3273593

[158] Orillion A , Hashimoto A , Damayanti N , et al. Entinostat neutralizes myeloid‐derived suppressor cells and enhances the antitumor effect of PD‐1 inhibition in murine models of lung and renal cell carcinoma. Clin Cancer Res. 2017;23:5187‐5201. doi:10.1158/1078-0432.CCR-17-0741 28698201 PMC5723438

[159] Flores‐Toro JA , Luo D , Gopinath A , et al. CCR2 inhibition reduces tumor myeloid cells and unmasks a checkpoint inhibitor effect to slow progression of resistant murine gliomas. Proc Natl Acad Sci U S A. 2020;117:1129‐1138. doi:10.1073/pnas.1910856117 31879345 PMC6969504

[160] Feins S , Kong W , Williams EF , Milone MC , Fraietta JA . An introduction to chimeric antigen receptor (CAR) T‐cell immunotherapy for human cancer. Am J Hematol. 2019;94:S3‐S9. doi:10.1002/ajh.25418 30680780

[161] June CH , Sadelain M . Chimeric antigen receptor therapy. N Engl J Med. 2018;379:64‐73. doi:10.1056/NEJMra1706169 29972754 PMC7433347

[162] Klichinsky M , Ruella M , Shestova O , et al. Human chimeric antigen receptor macrophages for cancer immunotherapy. Nat Biotechnol. 2020;38:947‐953. doi:10.1038/s41587-020-0462-y 32361713 PMC7883632

[163] Mayadas TN , Cullere X , Lowell CA . The multifaceted functions of neutrophils. Annu Rev Pathol. 2014;9:181‐218. doi:10.1146/annurev-pathol-020712-164023 24050624 PMC4277181

[164] Blanter M , Gouwy M , Struyf S . Studying neutrophil function in vitro: cell models and environmental factors. J Inflamm Res. 2021;14:141‐162. doi:10.2147/JIR.S284941 33505167 PMC7829132

[165] Gungabeesoon J , Gort‐Freitas NA , Kiss M , et al. A neutrophil response linked to tumor control in immunotherapy. Cell. 2023;186:1448‐1464.e20. doi:10.1016/j.cell.2023.02.032 37001504 PMC10132778

[166] Hirschhorn D , Budhu S , Kraehenbuehl L , et al. T cell immunotherapies engage neutrophils to eliminate tumor antigen escape variants. Cell. 2023;186:1432‐1447.e17. doi:10.1016/j.cell.2023.03.007 37001503 PMC10994488

[167] Hirschhorn‐Cymerman D , Budhu S , Kitano S , et al. Induction of tumoricidal function in CD4+ T cells is associated with concomitant memory and terminally differentiated phenotype. J Exp Med. 2012;209:2113‐2126. doi:10.1084/jem.20120532 23008334 PMC3478933

